# A Tandem Robotic Arm Inverse Kinematic Solution Based on an Improved Particle Swarm Algorithm

**DOI:** 10.3389/fbioe.2022.832829

**Published:** 2022-05-19

**Authors:** Guojun Zhao, Du Jiang, Xin Liu, Xiliang Tong, Ying Sun, Bo Tao, Jianyi Kong, Juntong Yun, Ying Liu, Zifan Fang

**Affiliations:** ^1^ Key Laboratory of Metallurgical Equipment and Control Technology of Ministry of Education, Wuhan University of Science and Technology, Wuhan, China; ^2^ Research Center for Biomimetic Robot and Intelligent Measurement and Control, Wuhan University of Science and Technology, Wuhan, China; ^3^ Hubei Key Laboratory of Mechanical Transmission and Manufacturing Engineering, Wuhan University of Science and Technology, Wuhan, China; ^4^ Precision Manufacturing Research Institute, Wuhan University of Science and Technology, Wuhan, China; ^5^ Hubei Key Laboratory of Hydroelectric Machinery Design & Maintenance, China Three Gorges University, Yichang, China

**Keywords:** particle swarm algorithm, joint limiting, adaptive strategy, spinor theory, robot inverse kinematics solution

## Abstract

The analysis of robot inverse kinematic solutions is the basis of robot control and path planning, and is of great importance for research. Due to the limitations of the analytical and geometric methods, intelligent algorithms are more advantageous because they can obtain approximate solutions directly from the robot’s positive kinematic equations, saving a large number of computational steps. Particle Swarm Algorithm (PSO), as one of the intelligent algorithms, is widely used due to its simple principle and excellent performance. In this paper, we propose an improved particle swarm algorithm for robot inverse kinematics solving. Since the setting of weights affects the global and local search ability of the algorithm, this paper proposes an adaptive weight adjustment strategy for improving the search ability. Considering the running time of the algorithm, this paper proposes a condition setting based on the limit joints, and introduces the position coefficient k in the velocity factor. Meanwhile, an exponential product form modeling method (POE) based on spinor theory is chosen. Compared with the traditional DH modeling method, the spinor approach describes the motion of a rigid body as a whole and avoids the singularities that arise when described by a local coordinate system. In order to illustrate the advantages of the algorithm in terms of accuracy, time, convergence and adaptability, three experiments were conducted with a general six-degree-of-freedom industrial robotic arm, a PUMA560 robotic arm and a seven-degree-of-freedom robotic arm as the research objects. In all three experiments, the parameters of the robot arm, the range of joint angles, and the initial attitude and position of the end-effector of the robot arm are given, and the attitude and position of the impact point of the end-effector are set to verify whether the joint angles found by the algorithm can reach the specified positions. In Experiments 2 and 3, the algorithm proposed in this paper is compared with the traditional particle swarm algorithm (PSO) and quantum particle swarm algorithm (QPSO) in terms of position and direction solving accuracy, operation time, and algorithm convergence. The results show that compared with the other two algorithms, the algorithm proposed in this paper can ensure higher position accuracy and orientation accuracy of the robotic arm end-effector. the position error of the algorithm proposed in this paper is 0 and the maximum orientation error is 1.29 × 10^–8^. while the minimum position error of the other two algorithms is −1.64 × 10^–5^ and the minimum orientation error is −4.03 × 10^–6^. In terms of operation time, the proposed algorithm in this paper has shorter operation time compared with the other two algorithms. In the last two experiments, the computing time of the proposed algorithm is 0.31851 and 0.30004s respectively, while the shortest computing time of the other two algorithms is 0.33359 and 0.30521s respectively. In terms of algorithm convergence, the proposed algorithm can achieve faster and more stable convergence than the other two algorithms. After changing the experimental subjects, the proposed algorithm still maintains its advantages in terms of accuracy, time and convergence, which indicates that the proposed algorithm is more applicable and has certain potential in solving the multi-arm inverse kinematics solution. This paper provides a new way of thinking for solving the multi-arm inverse kinematics solution problem.

## 1 Introduction

For the trajectory planning as well as control of the robotic arm, its inverse kinematic solution is the key. The inverse kinematic solution can directly affect the control accuracy and the success of trajectory planning of the robot arm. However, the process of solving the inverse kinematic solution for the robot arm is not only tedious, but also impossible to solve. The emergence of bionic intelligent algorithms provides new ideas for solving inverse kinematics solutions. By transforming the tedious inverse kinematics solution process into an optimization problem with minimum value, it not only simplifies the solution process, but also improves the solution efficiency. The particle swarm algorithm is more advantageous in terms of accuracy, speed and applicability at the level of robot inverse kinematics solution due to its simple programming and easy implementation. Therefore, this paper selects the particle swarm algorithm and further improves it for solving the robot inverse kinematics solution.

In this paper, an inverse kinematic solution method based on an improved particle swarm algorithm is proposed for the inverse kinematic solution of an arbitrary tandem robotic arm, with the following innovative points.

1) A particle swarm algorithm is introduced to solve the inverse kinematic solution of the tandem multi-degree-of-freedom robotic arm, which transforms the inverse kinematic solution process of the robotic arm into a multi-objective optimization problem and gives a suitable fitness function based on the inverse kinematic problem.
2) In this paper, an exponential product form modeling method (POE) based on spinor theory is chosen. Compared with the traditional DH modeling method, the spinor approach describes the motion of a rigid body as a whole and avoids the singularities that arise when described by a local coordinate system.
3) This paper proposes a condition setting based on limit joints and introduces a position factor k in the velocity factor. The reasonable condition setting provides a reference standard for the initialization of position and velocity, and reduces the running time of the algorithm at the same time. Among them, the operation time of the algorithm proposed in this paper is 0.31851 and 0.30004s in the second as well as the third experiment, while the shortest operation time of the other two algorithms is 0.33359 and 0.30521s, respectively.
4) An adaptive weight adjustment strategy is proposed to improve the stable search capability of the algorithm.
5) Three experiments are designed to illustrate the solution accuracy, operation time, and convergence of the algorithm. The experimental objects include: general six-degree-of-freedom robotic arm, PUMA560 robotic arm, and 7-degree-of-freedom robotic arm. The experimental method is: setting the position of the impact point and the attitude of the end-effector of the robotic arm, by bringing the relevant parameters of the robotic arm, the value range of the joint angle, the initial attitude and position of the end-effector of the robotic arm into the algorithm, finding the joint angle that meets the conditions, and finally getting the actual position and attitude of the end-effector of the robotic arm. The comparison algorithms include: the algorithm proposed in this paper, the traditional particle swarm algorithm (PSO), and the quantum particle swarm algorithm (QPSO). The comparison is done by comparing the error of the actual position and attitude of the robotic arm end-effector with the position and attitude of the given impact point, comparing the computing time of a set of data, and comparing the convergence of different algorithms according to the change of the fitness function with the number of iterations in a set of data. The results show that compared with the other two algorithms, the algorithm proposed in this paper can ensure higher position accuracy and orientation accuracy of the robotic arm end-effector. the position error of the algorithm proposed in this paper is 0 and the maximum orientation error is 1.29 × 10^–8^. while the minimum position error of the other two algorithms is −1.64 × 10^–5^ and the minimum orientation error is −4.03 × 10^–6^. In terms of operation time, the proposed algorithm in this paper has shorter operation time compared with the other two algorithms. In the last two experiments, the computing time of the proposed algorithm is 0.31851 and 0.30004s respectively, while the shortest computing time of the other two algorithms is 0.33359 and 0.30521s respectively. In terms of algorithm convergence, the proposed algorithm can achieve faster and more stable convergence than the other two algorithms.

The rest of this paper is described as follows. Section 2 introduces the current domestic and foreign methods for the inverse kinematics solution of robotic arms, the improvement methods of particle swarm algorithms, and the advantages and improvements of particle swarm algorithms for the inverse kinematics solution of robotic arms. Section 3 takes a general six-degree-of-freedom industrial robotic arm as the research object and analyzes its positive kinematics based on the spin volume theory, and also gives the specific calculation steps. Section 4 introduces two particle swarm optimization algorithms, explains the implementation steps of the general particle swarm algorithm, and illustrates the improvements of the algorithm compared with other improved particle swarm algorithms. Section 5 sets the experimental conditions and gives the specific form of the fitness function, the flowchart of the algorithm, and the pseudo-code. Section 6 conducts three experiments for different research objects, shows the simulation results under different conditions, and compares this algorithm with other algorithms in terms of solution accuracy, operation time, and convergence. Finally, the discussion and conclusion sections of this paper are presented.

## 2 Related Work

The main types of robot inverse kinematics solutions are analytical, numerical, geometric, and intelligent algorithms. The analytical method is mainly used to solve the robotic arm with a definite configuration, i.e., a robotic arm that satisfies the “Pieper” criterion - three joint axes intersecting at one point (Tong et al., 2021; Liu F. et al., 2021; Xiao et al., 2021; Chen et al., 2021a). When the criterion is satisfied, the joint angles of the robotic arm have a definite analytical solution form. The advantage of the analytical method is that it is fast to solve, and the disadvantage is that it has a more limited use. The numerical method has a wider application compared to the analytical method, but the solution speed is slow and there are numerical stability problems. The numerical method is based on the Jacobi matrix and approximates the optimal solution by numerical iteration. The geometric method has a narrower application than the analytical method. This method solves the inverse kinematic solution of the robotic arm mainly by the geometric configuration of the robotic arm. The robotic arm that does not satisfy the “Pieper” criterion can often be solved by the geometric method, and specific applications include the inverse kinematic solution of the three subproblems of “Paden-Kahan” based on the rotation theory (Wang et al., 2021; Weng et al., 2021; Duan et al., 2021; Cheng et al., 2021). The advantage of the geometric method is that it has a clear geometric meaning and can solve some problems that cannot be solved by the analytical method. Compared with the first two methods, the intelligent algorithm does not involve the inverse kinematic solution part, and solves the inverse kinematic solution mainly by deriving the end position change matrix based on the positive kinematics of the robot, and finally approximating the correct joint angle gradually by randomly generating the joint angle values and error functions to achieve the solution of the inverse kinematic solution. Intelligent algorithms tend to avoid some of the problems present in the process of solving conventional inverse kinematics solutions, such as the existence of singularities when the Jacobi determinant is zero, which cannot be solved (Kucuk and Bingul, 2014; Liao et al., 2021; Jiang et al., 2021a; Liu X. et al., 2021). Therefore it is of great research significance for the study of intelligent algorithms. The existing intelligent algorithms are artificial neural networks, adaptive neuro-fuzzy inference systems, and genetic algorithms, particle swarm search algorithms, etc. (EI-Sherbiny et al., 2018; Huang et al., 2021; Tao et al., 2021; Hao et al., 2021a).

Particle swarm algorithm is widely used as an intelligent algorithm compared to other algorithms because of its simple programming and easy implementation, as well as its better final solution. Many researchers have improved the particle swarm algorithm. Netjinda et al. optimized the search diversity of PSO algorithm by re-updating the position and velocity based on the principle of bird flock frightening (Netjinda et al., 2015; Jiang et al., 2021b; Ma et al., 2020; Sun et al., 2020a). Yang et al. improve the convergence of the algorithm generate the initial population by Halton sequence and adjust the inertia weights based on the variation property of the nonlinear function (Yang et al., 2015; Sun et al., 2020b; Luo et al., 2020; Sun et al., 2020c). Harrison et al. further study on the inertia weight adjustment strategy of particle swarm algorithm (Harrison et al., 2016; Li et al., 2020; Tan et al., 2020; Huang et al., 2020). Chen et al. proposed a double cluster and double layer structure, with the best particles as the top layer and all particles as the bottom layer to improve the search ability and efficiency of the algorithm (Chen et al., 2017; He et al., 2019; Jiang et al., 2019a; Chen et al., 2021b). Tanweer et al. divided the particle swarm into three groups, each with a different speed configuration. The speed of each group is configured with a different update strategy so that the algorithm achieves faster convergence and higher accuracy (Tanweer et al., 2016; Huang et al., 2019; Jiang et al., 2019b; Yu et al., 2019). Li et al. combine the particle swarm optimization algorithm with the artificial bee colony algorithm to improve the search capability and convergence speed of the algorithm (Li et al., 2015a; Sun et al., 2021; Tao et al., 2022a; Li et al., 2019b; Jiang et al., 2019c). Aydilek combined particle swarm optimization algorithm with firefly algorithm to improve the running time and convergence accuracy of the algorithm (Aydilek, 2018; Liu et al., 2021c; Liu et al., 2021d; Bai et al., 2022). Ngo et al. proposed a particle movement strategy for overcoming the situation that traditional particle swarm algorithms converge too early and fall into local optimum (Ngo et al., 2016; Liu et al., 2022d; Yang et al., 2021; Mao et al., 2017). Thangaraj et al. summarized the fusion algorithm of particle swarm algorithm with various other intelligent algorithms and conducted an experimental comparison (Thangaraj et al., 2011; Yang et al., 2021; Hao et al., 2021b; Tao et al., 2022b). Wei et al. proposed an adaptive two-layer particle swarm algorithm based on learning strategy by dividing the population into two parts (Lim and Mat Isa, 2014; Liu et al., 2022a; Yun et al., 2022; Liu et al., 2022b). Taherkhani et al. determines the inertia weight for each position based on the distance of each particle’s performance from the optimal position and ultimately improves the solution quality as well as the convergence speed (Taherkhani and Safabakhsh, 2016; Cheng et al., 2020; Wu et al., 2022; Yu et al., 2020).

For robot inverse kinematics solution solving, particle swarm algorithm is more advantageous in terms of accuracy, speed and applicability. Ayyildiz et al. compared genetic algorithm, particle swarm algorithm, quantum particle swarm algorithm and gravitational search algorithm for solving robot inverse kinematics solution and finally found that particle swarm algorithm has higher accuracy compared to other algorithms (Ayyildiz and Centinkaya, 2016). Dereli et al. proposed an improved PSO algorithm which discarded the traditional position and velocity update approach and chose to use a quantum mechanics based position update approach for solving the seven degree of freedom robotic arm inverse kinematics solution (Dereli and Koker, 2020). Liu et al. proposed an improved PSO algorithm for simultaneous optimization of multiple populations to enhance the search capability during population iteration (Liu F. et al., 2021; Chen et al., 2022; Sun et al., 2020d). Deng et al. proposed an adaptive particle swarm algorithm by improving the learning factor, adopting an adaptive weighting strategy, and proposing a special boundary handling method thereby optimizing the case where the particles fall into local optima (Deng and Xie, 2021; Xu et al., 2022; Sun et al., 2020a). Dereli et al. changed the fixed weights to variable random weights, while applying the improved PSO algorithm to the estimation of the end position of a seven-degree-of-freedom redundant robotic arm, ultimately improving its solution accuracy (Dereli and Koker, 2018). Pathak et al. proposed a bi-directional particle swarm optimization algorithm for solving the optimization problem of the inverse kinematic solution of a robotic arm (Pathak et al., 2019; Li et al., 2015b). Liu et al. proposed a new parallel learning particle swarm optimization algorithm (PLPSO) that divides the original population into two independently evolving subpopulations. The algorithm was compared with other algorithms and tested for the UR5 robotic arm, which finally showed the good performance of the algorithm (Liu et al., 2022c; Yiyang et al., 2021; Sun et al., 2018). Shastri et al., 2019 combined neural network with particle swarm algorithm to solve the robotic arm inverse kinematics solution from the operation time and complexity level (Shastri et al., 2019; Li et al., 2019c; Liu et al., 2022d; Li et al., 2019a).

## 3 Model and Kinematic Analysis

### 3.1 Robotic Arm Model

**FIGURE 1:**
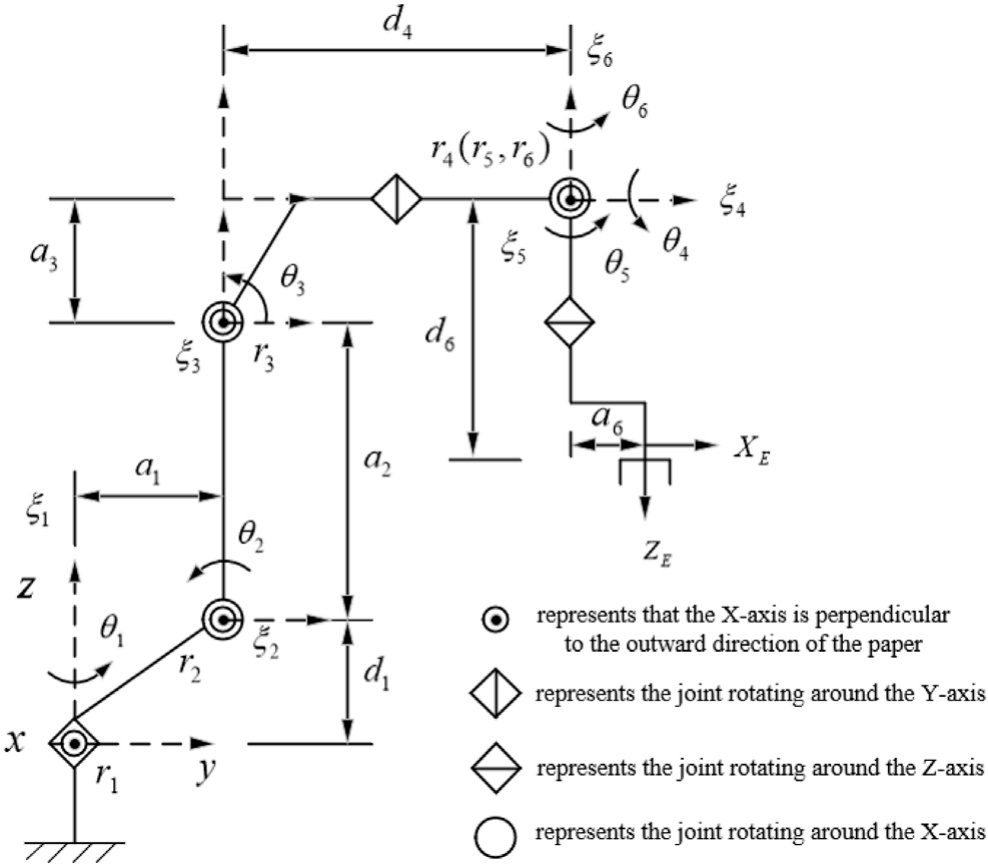
Sketch of general industrial six-degree-of-freedom robotic arm structure.

In this paper, a common six-degree-of-freedom robotic arm in industry is selected as the research object, and its structural sketch is shown in Figure 1. The robotic arm shown in Figure 1 is a six-degree-of-freedom tandem robotic arm, and all six joints are rotating joints. Its structure is characterized by the first three joints not intersecting, the second and third joint axes are parallel and anisotropic to the first joint axis, and the fourth, fifth and sixth joints intersect at a point, satisfying Pieper’s criterion, so there exists an inverse kinematic closure solution. Considering that the process of establishing the coordinate system by DH kinematic modeling method is too complicated, not only the world coordinate system needs to be established, but also the relative coordinate system between joints and joints, and the form of the final solution obtained is often inconsistent for different DH modeling methods. While based on the spinor theory only needs to establish a world coordinate system, which not only optimizes the modeling process, but also has good geometric meaning. Therefore, in this paper, we choose to establish the joint coordinate system based on the Lie group and spinor theory (Wang et al., 2018; Liao et al., 2020; Sun and Zhao, 2022; Zhang et al., 2022).

### 3.2 Kinematic Analysis

Compared with the traditional DH modeling method, the spinor approach describes the motion of a rigid body as a whole, avoiding the singularities that arise when described by a local coordinate system. One of the positive kinematic modeling processes based on the spinor theory is shown below.

The coordinates of a rigid body in space can be expressed as the transformation of the pose of the rigid body with respect to the base coordinate system as well as the transformation of the position. The specific representation is shown in Eq. 1.

$$\begin{array}{l} SE\left(3\right)=\left\{\left[\begin{array}{cc} R & t\\ 0 & 1 \end{array}\right]\right\};R\in R^{3\times 3},\\ t\in R^{3},R^{T}R=I,\det\left(R\right)=1, \end{array}\}$$ (1)

where R is a 3-by-3 matrix representing the pose transformation of the rigid body with respect to the base coordinate system. t is a 3-by-1 column vector representing the position transformation of the rigid body with respect to the base coordinate system. Chasles’ theorem proves that the rigid body motion of any object from one position pattern to another can be realized by a compound rotation around a certain line and a movement along that line, and that the compound motion is called spiral motion, whose infinitesimal quantity is the element of the Lie algebra, i.e., the kinematic spin. the Lie algebra of SE(3) is denoted as se(3), where the elements are defined as follows:

$$\overset{\wedge }{\xi }=\left[\begin{array}{cc} \overset{\wedge }{\omega } & v\\ 0 & 0 \end{array}\right]={\left[\omega^{T};v^{T}\right]}^{T}\in R^{6}$$ (2)

where ω denotes the angular velocity of rotation of the rigid body around the rotation axis. v denotes the corresponding translational velocity of the rigid body. According to the rotation theory, if the spiral motion of a rigid body is known, the linkage position and attitude transformation matrix can be expressed in the form of an exponential product (POE). The specific form is shown in Eq. 3.

$${\text{e}}^{\xi \theta }=\left\{ \begin{array}{l} \left[\begin{array}{cc} e^{\overset{\wedge }{\omega }\theta } & \left(I-e^{\overset{\wedge }{\omega }\theta }\right)\left(\omega \times \nu \right)+\theta \omega \omega^{T}\nu \\ 0 & 1 \end{array}\right],\omega \neq 0\\ \left[\begin{array}{cc} I & \theta \nu \\ 0 & 1 \end{array}\right],\omega =0 \end{array}\right.$$ (3)

The above equation represents the posture transformation matrix corresponding to the joint angle when the rigid body is in spiral motion. where $e^{\overset{\wedge }{\omega }\theta }$ , I denotes attitude change and $\left(I-e^{\overset{\wedge }{\omega }\theta }\right)\left(\omega \times \nu \right)+\theta \omega \omega^{T}\nu$ , θν denotes position change. ω denotes the angular velocity of rotation of the rigid body around the rotation axis. v denotes the corresponding translational velocity of the rigid body. θ indicates the joint angle. The positive kinematic expression of the robot is obtained by multiplying the initial positional matrix with the transformation matrix corresponding to each joint angle, provided that the initial position, pose and each joint angle of the end-effector of the robot arm are known. Based on the spinor theory, the transformation matrix corresponding to each joint angle can be replaced by the form of exponential product (POE). The specific positive kinematic expression is shown in Eq. 4.

$$e=e^{\xi_{1}\theta_{1}}e^{\xi_{2}\theta_{2}}e^{\xi_{3}\theta_{3}}e^{\xi_{4}\theta_{4}}e^{\xi_{5}\theta_{5}}e^{\xi_{6}\theta_{6}}e_{0}$$ (4)

Where $e_{0}$ represents the initial position, attitude matrix of the end-effector of the robot arm. $e^{\xi_{1}\theta_{1}}$ represents the transformation matrix corresponding to joint angle 1, $e^{\xi_{2}\theta_{2}}$ represents the transformation matrix corresponding to joint angle 2, $e^{\xi_{3}\theta_{3}}$ represents the transformation matrix corresponding to joint angle 3, $e^{\xi_{4}\theta_{4}}$ represents the transformation matrix corresponding to joint angle 4, $e^{\xi_{5}\theta_{5}}$ represents the transformation matrix corresponding to joint angle 5, and $e^{\xi_{6}\theta_{6}}$ represents the transformation matrix corresponding to joint angle 6. e represents the final position, attitude matrix of the end-effector of the robot arm. The specific calculation process of the positive kinematics of the six-degree-of-freedom industrial robotic arm (Figure 1) based on the rotational volume theory is as follows.

Step 1: Determine the angular speed of rotation $\omega_{i}$ of each linkage (i = 1, 2, 3, 4, 5, 6).

$$\omega_{1}=\left[\begin{array}{c} 0\\ 0\\ 1 \end{array}\right],\omega_{2}=\left[\begin{array}{c} 1\\ 0\\ 0 \end{array}\right],\omega_{3}=\left[\begin{array}{c} 1\\ 0\\ 0 \end{array}\right],\omega_{4}=\left[\begin{array}{c} 0\\ 1\\ 0 \end{array}\right],\omega_{5}=\left[\begin{array}{c} 1\\ 0\\ 0 \end{array}\right],\omega_{6}=\left[\begin{array}{c} 0\\ 0\\ 1 \end{array}\right]$$

Step 2: Determine the position ${\text{r}}_{\text{i}}$ of each linkage (i = 1, 2, 3, 4, 5, 6).

$$r_{1}=\left[\begin{array}{c} 0\\ 0\\ 0 \end{array}\right],r_{2}=\left[\begin{array}{c} 0\\ a_{1}\\ d_{1} \end{array}\right],r_{3}=\left[\begin{array}{c} 0\\ a_{1}\\ d_{1}+a_{2} \end{array}\right],r_{4}=r_{5}=r_{6}=\left[\begin{array}{c} 0\\ a_{1}+d_{4}\\ d_{1}+a_{2}+a_{3} \end{array}\right]$$

Where a1, a2, a3, d1, d4 are the relevant parameters for a general six-degree-of-freedom industrial robotic arm.

Step 3: Determine the intermediate parameters $\overset{\wedge }{\omega_{i}}$ of each linkage (i = 1, 2, 3, 4, 5, 6).

$$\begin{array}{l} \begin{array}{ccc} \overset{\wedge }{\omega_{1}}=\left[\begin{array}{ccc} 0 & -1 & 0\\ 1 & 0 & 0\\ 0 & 0 & 0 \end{array}\right] & \overset{\wedge }{\omega_{2}}=\left[\begin{array}{ccc} 0 & 0 & 0\\ 0 & 0 & -1\\ 0 & 1 & 0 \end{array}\right] & \overset{\wedge }{\omega_{3}}=\left[\begin{array}{ccc} 0 & 0 & 0\\ 0 & 0 & -1\\ 0 & 1 & 0 \end{array}\right] \end{array}\\ \begin{array}{ccc} \overset{\wedge }{\omega_{4}}=\left[\begin{array}{ccc} 0 & 0 & -1\\ 0 & 0 & 0\\ 1 & 0 & 0 \end{array}\right] & \overset{\wedge }{\omega_{5}}=\left[\begin{array}{ccc} 0 & 0 & 0\\ 0 & 0 & -1\\ 0 & 1 & 0 \end{array}\right] & \overset{\wedge }{\omega_{6}}=\left[\begin{array}{ccc} 0 & -1 & 0\\ 1 & 0 & 0\\ 0 & 0 & 0 \end{array}\right] \end{array} \end{array}$$

Step 4: Determine the translational speed $v_{\text{i}}$ of each linkage (i = 1, 2, 3, 4, 5, 6).

$$v_{i}=-\omega_{i}\times r_{i}\;\;\;\left(\text{i}=1\text{, }2\text{, }3\text{, }4\text{, }5\text{, }6\right)$$ (5)

Step 4: Determine the attitude change matrix $R_{i}$ for each linkage (i = 1, 2, 3, 4, 5, 6).

$$R_{i}=\left[\begin{array}{ccc} 1 & 0 & 0\\ 0 & 1 & 0\\ 0 & 0 & 1 \end{array}\right]+\overset{\wedge }{\omega_{i}}*\sin\left(\theta_{i}\right)+\overset{\wedge }{\omega_{i}^{2}}*\left(1-\cos\left(\theta_{i}\right)\right)\;\;\left(\text{i}=1\text{, }2\text{, }3\text{, }4\text{, }5\text{, }6\right)$$ (6)

where $\theta_{i}$ represents the joint angle of the corresponding linkage i.

Step 6: Determine the position change matrix $t_{i}$ for each linkage (i = 1, 2, 3, 4, 5, 6).

$$t_{i}=\left(\left[\begin{array}{ccc} 1 & 0 & 0\\ 0 & 1 & 0\\ 0 & 0 & 1 \end{array}\right]-R_{i}\right)*\left(\omega_{i}\times \nu_{i}\right)+\omega_{i}*\omega_{i}^{T}*\nu_{i}*\theta_{i}\;\;\left(\text{i}=1\text{, }2\text{, }3\text{, }4\text{, }5\text{, }6\right)$$ (7)

Step 7: Determine the position, attitude change matrix ${\text{e}}^{\xi_{i}\theta_{i}}$ of each linkage (i = 1, 2, 3, 4, 5, 6).

$${\text{e}}^{\xi_{i}\theta_{i}}=\left[\begin{array}{cc} R_{i} & t_{i}\\ 0 & 1 \end{array}\right]\;\;\left(\text{i}=1\text{, }2\text{, }3\text{, }4\text{, }5\text{, }6\right)$$ (8)

Step 8: Find the robotic arm end-effector end position, attitude matrix e_0_ with the known robotic arm end-effector initial end position and attitude matrix e.

$$e=e^{\xi_{1}\theta_{1}}e^{\xi_{2}\theta_{2}}e^{\xi_{3}\theta_{3}}e^{\xi_{4}\theta_{4}}e^{\xi_{5}\theta_{5}}e^{\xi_{6}\theta_{6}}e_{0}$$ (9)

Among them, the positive kinematic solution idea for the position coordinates of the robotic arm end-effector and Euler angles of rotation along the *x*,*y*,*z* axes is as follows: first set the initial position coordinates of the robotic arm end-effector and Euler angles of rotation along the *x*,*y*,*z* axes to get the initial end position pose matrix of the robotic arm end-effector. Then the kinematic analysis of the robot arm is carried out to obtain the position pose change matrix of the end-effector of the robot arm, and finally the actual position pose matrix of the end-effector of the robot arm is obtained by multiplying the position pose change matrix with the initial position pose matrix. According to the actual position pose matrix, the actual position coordinates of the end-effector of the robot arm and Euler angles of rotation along the *x*,*y*,*z* axes can be obtained. The whole process realizes the transformation of the robotic arm from one position pose to another position pose. Among them, the initial position pose matrix of the robotic arm end-effector in experiment 1, the associated change matrix and Euler angles of rotation along the *x*,*y*,*z* axes of the end-effector, and the position expressions are shown below.

$$g=\left[\begin{array}{cccc} 1 & 0 & 0 & 0\\ 0 & 1 & 0 & a_{1}+{\mathrm{d}}_{4}+a_{6}\\ 0 & 0 & 1 & {\mathrm{d}}_{1}+a_{2}+a_{3}-{\mathrm{d}}_{6}\\ 0 & 0 & 0 & 1 \end{array}\right]$$ (10)

$$\begin{array}{l} e^{\xi_{1}\theta_{1}}=\left[\begin{array}{cccc} c_{1} & -s_{1} & 0 & 0\\ s_{1} & c_{1} & 0 & 0\\ 0 & 0 & 1 & 0\\ 0 & 0 & 0 & 1 \end{array}\right]\;e^{\xi_{2}\theta_{2}}=\left[\begin{array}{cccc} 1 & 0 & 0 & 0\\ 0 & c_{2} & -s_{2} & d_{1}s_{2}-a_{1}\left(c_{2}-1\right)\\ 0 & s_{2} & c_{2} & -a_{1}s_{2}-d_{1}\left(c_{2}-1\right)\\ 0 & 0 & 0 & 1 \end{array}\right]\\ e^{\xi_{3}\theta_{3}}=\left[\begin{array}{cccc} 1 & 0 & 0 & 0\\ 0 & c_{3} & -s_{3} & s_{3}\left(a_{2}+d_{1}\right)-a_{1}\left(c_{3}-1\right)\\ 0 & s_{3} & c_{3} & -a_{1}s_{3}-\left(a_{2}+d_{1}\right)\left(c_{3}-1\right)\\ 0 & 0 & 0 & 1 \end{array}\right]\;e^{\xi_{4}\theta_{4}}=\left[\begin{array}{cccc} c_{4} & 0 & -s_{4} & s_{4}\left(a_{2}+a_{3}+d_{1}\right)\\ 0 & 1 & 0 & 0\\ s_{4} & 0 & c_{4} & -\left(c_{4}-1\right)\left(a_{2}+a_{3}+d_{1}\right)\\ 0 & 0 & 0 & 1 \end{array}\right]\\ e^{\xi_{5}\theta_{5}}=\left[\begin{array}{cccc} 1 & 0 & 0 & 0\\ 0 & c_{5} & -s_{5} & s_{5}\left(a_{2}+a_{3}+d_{1}\right)-\left(a_{1}+d_{4}\right)\left(c_{5}-1\right))\\ 0 & s_{5} & c_{5} & -\left(c_{5}-1\right)\left(a_{2}+a_{3}+d_{1}\right)-s_{5}\left(a_{1}+d_{4}\right)\\ 0 & 0 & 0 & 1 \end{array}\right]\\ e^{\xi_{6}\theta_{6}}=\left[\begin{array}{cccc} c_{6} & -s_{6} & 0 & s_{6}\left(a_{1}+d_{4}\right)\\ s_{6} & c_{6} & 0 & -\left(a_{1}+d_{4}\right)\left(c_{6}-1\right)\\ 0 & 0 & 1 & 0\\ 0 & 0 & 0 & 1 \end{array}\right] \end{array}$$ (11)

$$\alpha =atan2\left(\begin{array}{l} {\text{c}}_{6}\left({\text{c}}_{5}\left({\text{c}}_{2}{\text{s}}_{3}{\;+\text{ c}}_{3}{\text{s}}_{2}\right){\;+\text{ c}}_{4}{\text{s}}_{5}\left({\text{c}}_{2}{\text{c}}_{3}{\;-\text{ s}}_{2}{\text{s}}_{3}\right)\right){\;-\text{ s}}_{4}{\text{s}}_{6}\left({\text{c}}_{2}{\text{c}}_{3}{\;-\text{ s}}_{2}{\text{s}}_{3}\right){\text{, c}}_{4}{\text{c}}_{5}\left({\text{c}}_{2}{\text{c}}_{3}{\;-\text{ s}}_{2}{\text{s}}_{3}\right)\;\\ {-\text{ s}}_{5}\left({\text{c}}_{2}{\text{s}}_{3}{\;+\text{ c}}_{3}{\text{s}}_{2}\right) \end{array}\right)$$ (12)

$$\beta =atan2\left(\begin{array}{l} -\left[{\text{s}}_{6}\left({\text{c}}_{5}\left({\text{c}}_{2}{\text{s}}_{3}{\;+\text{ c}}_{3}{\text{s}}_{2}\right){\;+\text{ c}}_{4}{\text{s}}_{5}\left({\text{c}}_{2}{\text{c}}_{3}{\;-\text{ s}}_{2}{\text{s}}_{3}\right)\right){\;+\text{ c}}_{6}{\text{s}}_{4}\left({\text{c}}_{2}{\text{c}}_{3}{\;-\text{ s}}_{2}{\text{s}}_{3}\right)\right]\\ ,sqrt\left(\begin{array}{l} {\left[{\text{c}}_{6}\left({\text{c}}_{5}\left({\text{c}}_{2}{\text{s}}_{3}{+\text{ c}}_{3}{\text{s}}_{2}\right){\;+\text{ c}}_{4}{\text{s}}_{5}\left({\text{c}}_{2}{\text{c}}_{3}{\;-\text{ s}}_{2}{\text{s}}_{3}\right)\right){\;-\text{ s}}_{4}{\text{s}}_{6}\left({\text{c}}_{2}{\text{c}}_{3}{\;-\text{ s}}_{2}{\text{s}}_{3}\right)\right]}^{2}\\ +{\left[{\text{c}}_{4}{\text{c}}_{5}\left({\text{c}}_{2}{\text{c}}_{3}{\;-\text{ s}}_{2}{\text{s}}_{3}\right){\;-\text{ s}}_{5}\left({\text{c}}_{2}{\text{s}}_{3}{\;+\text{ c}}_{3}{\text{s}}_{2}\right)\right]}^{2} \end{array}\right) \end{array}\right)$$ (13)

$$\eta =atan2\left(\begin{array}{l} {\text{s}}_{6}\left({\text{c}}_{5}\left({\text{c}}_{1}{\text{c}}_{2}{\text{c}}_{3}\;-{\text{ c}}_{1}{\text{s}}_{2}{\text{s}}_{3}\right)\;-{\text{ s}}_{5}\left({\text{s}}_{1}{\text{s}}_{4}{\;+\text{ c}}_{4}\left({\text{c}}_{1}{\text{c}}_{2}{\text{s}}_{3}{\;+\text{ c}}_{1}{\text{c}}_{3}{\text{s}}_{2}\right)\right)\right)\\ {+\text{ c}}_{6}\left({\text{c}}_{4}{\text{s}}_{1}\;-{\text{ s}}_{4}\left({\text{c}}_{1}{\text{c}}_{2}{\text{s}}_{3}{+\text{ c}}_{1}{\text{c}}_{3}{\text{s}}_{2}\right)\right){\text{, c}}_{6}\left({\text{c}}_{1}{\text{c}}_{4}{\;+\text{ s}}_{4}\left({\text{c}}_{2}{\text{s}}_{1}{\text{s}}_{3}{\;+\text{ c}}_{3}{\text{s}}_{1}{\text{s}}_{2}\right)\right)\\ -{\text{ s}}_{6}\left({\text{c}}_{5}\left({\text{c}}_{2}{\text{c}}_{3}{\text{s}}_{1}\;-{\text{ s}}_{1}{\text{s}}_{2}{\text{s}}_{3}\right){+\text{ s}}_{5}\left({\text{c}}_{1}{\text{s}}_{4}\;-{\text{ c}}_{4}\left({\text{c}}_{2}{\text{s}}_{1}{\text{s}}_{3}{+\text{ c}}_{3}{\text{s}}_{1}{\text{s}}_{2}\right)\right)\right) \end{array}\right)$$ (14)

$$\begin{array}{l} P_{x}={\text{a}}_{2}{\text{s}}_{1}{\text{s}}_{2}-{\text{a}}_{1}{\text{s}}_{1}{\;+\text{ a}}_{3}{\text{c}}_{2}{\text{s}}_{1}{\text{s}}_{3}{\;+\text{ a}}_{3}{\text{c}}_{3}{\text{s}}_{1}{\text{s}}_{2}{\;+\text{ d}}_{4}{\text{s}}_{1}{\text{s}}_{2}{\text{s}}_{3}\;-{\text{ a}}_{6}{\text{c}}_{1}{\text{c}}_{4}{\text{s}}_{6}\;-{\text{ c}}_{2}{\text{c}}_{3}{\text{d}}_{4}{\text{s}}_{1}{\;+\text{ c}}_{1}{\text{c}}_{5}{\text{d}}_{6}{\text{s}}_{4}\;\\ -{\text{ a}}_{6}{\text{c}}_{1}{\text{c}}_{6}{\text{s}}_{4}{\text{s}}_{5}\;-{\text{ c}}_{2}{\text{c}}_{3}{\text{d}}_{6}{\text{s}}_{1}{\text{s}}_{5}{\;+\text{ d}}_{6}{\text{s}}_{1}{\text{s}}_{2}{\text{s}}_{3}{\text{s}}_{5}\;-{\text{ a}}_{6}{\text{c}}_{2}{\text{c}}_{3}{\text{c}}_{5}{\text{c}}_{6}{\text{s}}_{1}-{\text{ c}}_{2}{\text{c}}_{4}{\text{c}}_{5}{\text{d}}_{6}{\text{s}}_{1}{\text{s}}_{3}\;-{\text{ c}}_{3}{\text{c}}_{4}{\text{c}}_{5}{\text{d}}_{6}{\text{s}}_{1}{\text{s}}_{2}\;\\ {+\text{ a}}_{6}{\text{c}}_{5}{\text{c}}_{6}{\text{s}}_{1}{\text{s}}_{2}{\text{s}}_{3}\;-{\text{ a}}_{6}{\text{c}}_{2}{\text{s}}_{1}{\text{s}}_{3}{\text{s}}_{4}{\text{s}}_{6}\;-{\text{ a}}_{6}{\text{c}}_{3}{\text{s}}_{1}{\text{s}}_{2}{\text{s}}_{4}{\text{s}}_{6}{\;+\text{ a}}_{6}{\text{c}}_{2}{\text{c}}_{4}{\text{c}}_{6}{\text{s}}_{1}{\text{s}}_{3}{\text{s}}_{5}{\;+\text{ a}}_{6}{\text{c}}_{3}{\text{c}}_{4}{\text{c}}_{6}{\text{s}}_{1}{\text{s}}_{2}{\text{s}}_{5} \end{array}$$ (15)

$$\begin{array}{l} P_{y}=a_{1}c_{1}-a_{2}c_{1}s_{2}-a_{6}c_{4}s_{1}s_{6}-c_{1}d_{4}s_{2}s_{3}+c_{5}d_{6}s_{1}s_{4}+c_{1}c_{2}c_{3}d_{4}-a_{3}c_{1}c_{2}s_{3}-a_{3}c_{1}c_{3}s_{2}\\ +c_{1}c_{2}c_{3}d_{6}s_{5}-a_{6}c_{6}s_{1}s_{4}s_{5}-c_{1}d_{6}s_{2}s_{3}s_{5}+a_{6}c_{1}c_{2}c_{3}c_{5}c_{6}+c_{1}c_{2}c_{4}c_{5}d_{6}s_{3}+c_{1}c_{3}c_{4}c_{5}d_{6}s_{2}\\ -a_{6}c_{1}c_{5}c_{6}s_{2}s_{3}+a_{6}c_{1}c_{2}s_{3}s_{4}s_{6}+a_{6}c_{1}c_{3}s_{2}s_{4}s_{6}-a_{6}c_{1}c_{2}c_{4}c_{6}s_{3}s_{5}-a_{6}c_{1}c_{3}c_{4}c_{6}s_{2}s_{5} \end{array}$$ (16)

$$\begin{array}{l} P_{z}=d_{1}+a_{2}c_{2}+a_{3}c_{2}c_{3}+c_{2}d_{4}s_{3}+c_{3}d_{4}s_{2}-a_{3}s_{2}s_{3}+c_{2}d_{6}s_{3}s_{5}+c_{3}d_{6}s_{2}s_{5}-c_{2}c_{3}c_{4}c_{5}d_{6}\\ +a_{6}c_{2}c_{5}c_{6}s_{3}+a_{6}c_{3}c_{5}c_{6}s_{2}-a_{6}c_{2}c_{3}s_{4}s_{6}+c_{4}c_{5}d_{6}s_{2}s_{3}+a_{6}s_{2}s_{3}s_{4}s_{6}+a_{6}c_{2}c_{3}c_{4}c_{6}s_{5}-a_{6}c_{4}c_{6}s_{2}s_{3}s_{5} \end{array}$$ (17)

where $s_{i}=\sin\theta_{i}\left(i=1∼6\right)$ , $c_{i}=\cos\theta_{i}\left(i=1∼6\right)$ . $a_{1},a_{2},a_{3},a_{6},d_{1},d_{4},d_{6}$ denotes the parameters of a general six-degree-of-freedom industrial robot arm. g represents the initial position attitude matrix of the end-effector, $e^{\xi_{1}\theta_{1}}∼e^{\xi_{6}\theta_{6}}$ represents the variation matrix of the joint angles from 1 to 6, α,β,η represents Euler angles of rotation along the *x*,*y*,*z* axes after the end-effector moves from the initial position, and $P_{x},P_{y},P_{z}$ represents the new position of the end-effector after the end-effector moves from the initial position. The initial position pose matrix of the robotic arm end-effector in experiment 2, the associated change matrix and the three Euler angles of the end-effector, and the position expressions are shown below.

$$g=\left[\begin{array}{cccc} 0.9998 & 0.0175 & 0 & a_{2}+a_{3}\\ -0.0175 & 0.9998 & 0 & d_{2}\\ 0 & 0 & 1 & -d_{4}\\ 0 & 0 & 0 & 1 \end{array}\right]$$ (18)

$$\begin{array}{l} \begin{array}{ccc} T_{1}=\left[\begin{array}{cccc} c_{1} & -s_{1} & 0 & 0\\ s_{1} & c_{1} & 0 & 0\\ 0 & 0 & 1 & 0\\ 0 & 0 & 0 & 1 \end{array}\right] & T_{2}=\left[\begin{array}{cccc} c_{2} & -s_{2} & 0 & 0\\ 0 & 0 & 1 & d_{2}\\ -s_{2} & -c_{2} & 0 & 0\\ 0 & 0 & 0 & 1 \end{array}\right] & T_{3}=\left[\begin{array}{cccc} c_{3} & -s_{3} & 0 & a_{2}\\ s_{3} & c_{3} & 0 & 0\\ 0 & 0 & 1 & 0\\ 0 & 0 & 0 & 1 \end{array}\right] \end{array}\\ \begin{array}{ccc} T_{4}=\left[\begin{array}{cccc} c_{4} & -s_{4} & 0 & a_{3}\\ 0 & 0 & 1 & d_{4}\\ -s_{4} & -c_{4} & 0 & 0\\ 0 & 0 & 0 & 1 \end{array}\right] & T_{5}=\left[\begin{array}{cccc} c_{5} & -s_{5} & 0 & 0\\ 0 & 0 & -1 & 0\\ -s_{5} & -c_{5} & 0 & 0\\ 0 & 0 & 0 & 1 \end{array}\right] & T_{6}=\left[\begin{array}{cccc} c_{6} & -s_{6} & 0 & 0\\ 0 & 0 & 1 & 0\\ -s_{6} & -c_{6} & 0 & 0\\ 0 & 0 & 0 & 1 \end{array}\right] \end{array} \end{array}$$ (19)

$$\alpha =\mathrm{atan}2\left(\begin{array}{l} \left(4999s_{6}\left(s_{5}\left(c_{2}c_{3}-s_{2}s_{3}\right)+c_{4}c_{5}\left(c_{2}s_{3}+c_{3}s_{2}\right)\right)\right)/5000\\ -\left(7c_{6}\left(s_{5}\left(c_{2}c_{3}-s_{2}s_{3}\right)+c_{4}c_{5}\left(c_{2}s_{3}+c_{3}s_{2}\right)\right)\right)/400+\left(7s_{4}s_{6}\left(c_{2}s_{3}+c_{3}s_{2}\right)\right)/400\\ +\left(4999c_{6}s_{4}\left(c_{2}s_{3}+c_{3}s_{2}\right)\right)/5000,\\ c_{4}s_{5}\left(c_{2}s_{3}+c_{3}s_{2}\right)-c_{5}\left(c_{2}c_{3}-s_{2}s_{3}\right) \end{array}\right)$$ (20)

$$\beta =\mathrm{atan}2\left(\begin{array}{l} -\left[\begin{array}{l} \left(4999s_{4}s_{6}\left(c_{2}s_{3}+c_{3}s_{2}\right)\right)/5000-\left(7s_{6}\left(s_{5}\left(c_{2}c_{3}-s_{2}s_{3}\right)+c_{4}c_{5}\left(c_{2}s_{3}+c_{3}s_{2}\right)\right)\right)/400\\ -\left(4999c_{6}\left(s_{5}\left(c_{2}c_{3}-s_{2}s_{3}\right)+c_{4}c_{5}\left(c_{2}s_{3}+c_{3}s_{2}\right)\right)\right)/5000\\ -\left(7c_{6}s_{4}\left(c_{2}s_{3}+c_{3}s_{2}\right)\right)/400 \end{array}\right],\\ sqrt\left(\begin{array}{l} {\left[\begin{array}{l} \left(4999s_{6}\left(s_{5}\left(c_{2}c_{3}-s_{2}s_{3}\right)+c_{4}c_{5}\left(c_{2}s_{3}+c_{3}s_{2}\right)\right)\right)/5000\\ -\left(7c_{6}\left(s_{5}\left(c_{2}c_{3}-s_{2}s_{3}\right)+c_{4}c_{5}\left(c_{2}s_{3}+c_{3}s_{2}\right)\right)\right)/400\\ +\left(7s_{4}s_{6}\left(c_{2}s_{3}+c_{3}s_{2}\right)\right)/400+\left(4999c_{6}s_{4}\left(c_{2}s_{3}+c_{3}s_{2}\right)\right)/5000 \end{array}\right]}^{2}\\ +{\left[c_{4}s_{5}\left(c_{2}s_{3}+c_{3}s_{2}\right)-c_{5}\left(c_{2}c_{3}-s_{2}s_{3}\right)\right]}^{2} \end{array}\right) \end{array}\right)$$ (21)

$$\eta =\mathrm{atan}2\left(\begin{array}{l} \left(7c_{6}\left(c_{1}c_{4}+s_{4}\left(c_{2}c_{3}s_{1}-s_{1}s_{2}s_{3}\right)\right)\right)/400\\ -\left(7s_{6}\left(s_{5}\left(c_{2}s_{1}s_{3}+c_{3}s_{1}s_{2}\right)+c_{5}\left(c_{1}s_{4}-c_{4}\left(c_{2}c_{3}s_{1}-s_{1}s_{2}s_{3}\right)\right)\right)\right)/400\\ -\left(4999c_{6}\left(s_{5}\left(c_{2}s_{1}s_{3}+c_{3}s_{1}s_{2}\right)+c_{5}\left(c_{1}s_{4}-c_{4}\left(c_{2}c_{3}s_{1}-s_{1}s_{2}s_{3}\right)\right)\right)\right)/5000\\ -\left(4999s_{6}\left(c_{1}c_{4}+s_{4}\left(c_{2}c_{3}s_{1}-s_{1}s_{2}s_{3}\right)\right)\right)/5000\\ ,\left(4999s_{6}\left(c_{4}s_{1}-s_{4}\left(c_{1}c_{2}c_{3}-c_{1}s_{2}s_{3}\right)\right)\right)/5000\\ -\left(7s_{6}\left(s_{5}\left(c_{1}c_{2}s_{3}+c_{1}c_{3}s_{2}\right)-c_{5}\left(s_{1}s_{4}+c_{4}\left(c_{1}c_{2}c_{3}-c_{1}s_{2}s_{3}\right)\right)\right)\right)/400\\ -\left(7c_{6}\left(c_{4}s_{1}-s_{4}\left(c_{1}c_{2}c_{3}-c_{1}s_{2}s_{3}\right)\right)\right)/400\\ -\left(4999c_{6}\left(s_{5}\left(c_{1}c_{2}s_{3}+c_{1}c_{3}s_{2}\right)-c_{5}\left(s_{1}s_{4}+c_{4}\left(c_{1}c_{2}c_{3}-c_{1}s_{2}s_{3}\right)\right)\right)\right)/5000 \end{array}\right)$$ (22)

$$\begin{array}{l} P_{x}=d_{2}\left(s_{6}\left(s_{5}\left(c_{1}c_{2}s_{3}+c_{1}c_{3}s_{2}\right)-c_{5}\left(s_{1}s_{4}+c_{4}\left(c_{1}c_{2}c_{3}-c_{1}s_{2}s_{3}\right)\right)\right)+c_{6}\left(c_{4}s_{1}-s_{4}\left(c_{1}c_{2}c_{3}-c_{1}s_{2}s_{3}\right)\right)\right)\\ -d_{2}s_{1}+d_{4}\left(c_{5}\left(c_{1}c_{2}s_{3}+c_{1}c_{3}s_{2}\right)+s_{5}\left(s_{1}s_{4}+c_{4}\left(c_{1}c_{2}c_{3}-c_{1}s_{2}s_{3}\right)\right)\right)+a_{3}\left(c_{1}c_{2}c_{3}-c_{1}s_{2}s_{3}\right)\\ -d_{4}\left(c_{1}c_{2}s_{3}+c_{1}c_{3}s_{2}\right)-\left(a_{2}+a_{3}\right)\left(\begin{array}{l} c_{6}\left(s_{5}\left(c_{1}c_{2}s_{3}+c_{1}c_{3}s_{2}\right)-c_{5}\left(s_{1}s_{4}+c_{4}\left(c_{1}c_{2}c_{3}-c_{1}s_{2}s_{3}\right)\right)\right)\\ -s_{6}\left(c_{4}s_{1}-s_{4}\left(c_{1}c_{2}c_{3}-c_{1}s_{2}s_{3}\right)\right) \end{array}\right)+a_{2}c_{1}c_{2} \end{array}$$ (23)

$$\begin{array}{l} P_{y}=c_{1}d_{2}-\left(a_{2}+a_{3}\right)\left(c_{6}\left(s_{5}\left(c_{2}s_{1}s_{3}+c_{3}s_{1}s_{2}\right)+c_{5}\left(c_{1}s_{4}-c_{4}\left(c_{2}c_{3}s_{1}-s_{1}s_{2}s_{3}\right)\right)\right)+s_{6}\left(c_{1}c_{4}+s_{4}\left(c_{2}c_{3}s_{1}-s_{1}s_{2}s_{3}\right)\right)\right)\\ +d_{2}\left(s_{6}\left(s_{5}\left(c_{2}s_{1}s_{3}+c_{3}s_{1}s_{2}\right)+c_{5}\left(c_{1}s_{4}-c_{4}\left(c_{2}c_{3}s_{1}-s_{1}s_{2}s_{3}\right)\right)\right)-c_{6}\left(c_{1}c_{4}+s_{4}\left(c_{2}c_{3}s_{1}-s_{1}s_{2}s_{3}\right)\right)\right)\\ +d_{4}\left(c_{5}\left(c_{2}s_{1}s_{3}+c_{3}s_{1}s_{2}\right)-s_{5}\left(c_{1}s_{4}-c_{4}\left(c_{2}c_{3}s_{1}-s_{1}s_{2}s_{3}\right)\right)\right)+a_{3}\left(c_{2}c_{3}s_{1}-s_{1}s_{2}s_{3}\right)\\ -d_{4}\left(c_{2}s_{1}s_{3}+c_{3}s_{1}s_{2}\right)+a_{2}c_{2}s_{1} \end{array}$$ (24)

$$\begin{array}{l} P_{z}={\text{d}}_{4}\left({\text{c}}_{5}\left({\text{c}}_{2}{\text{c}}_{3}{\;-\text{ s}}_{2}{\text{s}}_{3}\right){\;-\text{ c}}_{4}{\text{s}}_{5}\left({\text{c}}_{2}{\text{s}}_{3}{\;+\text{ c}}_{3}{\text{s}}_{2}\right)\right){-\text{ a}}_{3}\left({\text{c}}_{2}{\text{s}}_{3}{\;+\text{ c}}_{3}{\text{s}}_{2}\right)\\ {-\text{ d}}_{4}\left({\text{c}}_{2}{\text{c}}_{3}{\;-\text{ s}}_{2}{\text{s}}_{3}\right)-\left({\text{c}}_{6}\left({\text{s}}_{5}\left({\text{c}}_{2}{\text{c}}_{3}{\;-\text{ s}}_{2}{\text{s}}_{3}\right){\;+\text{ c}}_{4}{\text{c}}_{5}\left({\text{c}}_{2}{\text{s}}_{3}{\;+\text{ c}}_{3}{\text{s}}_{2}\right)\right){-\text{ s}}_{4}{\text{s}}_{6}\left({\text{c}}_{2}{\text{s}}_{3}{\;+\text{ c}}_{3}{\text{s}}_{2}\right)\right)\left({\text{a}}_{2}{\;+\text{ a}}_{3}\right){\;-\text{ a}}_{2}{\text{s}}_{2}\\ {+\text{ d}}_{2}\left({\text{s}}_{6}\left({\text{s}}_{5}\left({\text{c}}_{2}{\text{c}}_{3}{\;-\text{ s}}_{2}{\text{s}}_{3}\right){+\text{ c}}_{4}{\text{c}}_{5}\left({\text{c}}_{2}{\text{s}}_{3}{\;+\text{ c}}_{3}{\text{s}}_{2}\right)\right){+\text{ c}}_{6}{\text{s}}_{4}\left({\text{c}}_{2}{\text{s}}_{3}{\;+\text{ c}}_{3}{\text{s}}_{2}\right)\right) \end{array}$$ (25)

where $s_{i}=\sin\theta_{i}\left(i=1∼6\right)$ , $c_{i}=\cos\theta_{i}\left(i=1∼6\right)$ . $a_{2},a_{3},d_{2},d_{4}$ denotes the parameters of PUMA560 robot arm. g represents the initial position attitude matrix of the end-effector, $T_{1}∼T_{6}$ represents the variation matrix of the joint angles from 1 to 6, α,β,η represents Euler angles of rotation along the *x*,*y*,*z* axes after the end-effector moves from the initial position, and $P_{x},P_{y},P_{z}$ represents the new position of the end-effector after the end-effector moves from the initial position. The initial position pose matrix of the robotic arm end-effector in experiment 3, the associated change matrix and the three Euler angles of the end-effector, and the position expressions are shown below.

$$g=\left[\begin{array}{cccc} 0.9998 & 0 & 0.0175 & a_{2}+a_{3}+a_{4}+a_{5}+a_{6}+a_{7}\\ 0 & 1 & 0 & d_{7}\\ -0.0175 & 0 & 0.9998 & d_{1}\\ 0 & 0 & 0 & 1 \end{array}\right]$$ (26)

$$\begin{array}{l} T_{1}=\left[\begin{array}{cccc} c_{1} & 0 & -s_{1} & 0\\ s_{1} & 0 & c_{1} & 0\\ 0 & -1 & 0 & d_{1}\\ 0 & 0 & 0 & 1 \end{array}\right]\;T_{2}=\left[\begin{array}{cccc} c_{2} & 0 & s_{2} & a_{2}c_{2}\\ s_{2} & 0 & -c_{2} & a_{2}s_{2}\\ 0 & 1 & 0 & 0\\ 0 & 0 & 0 & 1 \end{array}\right]\;T_{3}=\left[\begin{array}{cccc} c_{3} & 0 & -s_{3} & a_{3}c_{3}\\ s_{3} & 0 & c_{3} & a_{3}s_{3}\\ 0 & -1 & 0 & 0\\ 0 & 0 & 0 & 1 \end{array}\right]\\ T_{4}=\left[\begin{array}{cccc} c_{4} & 0 & s_{4} & a_{4}c_{4}\\ s_{4} & 0 & -c_{4} & a_{4}s_{4}\\ 0 & 1 & 0 & 0\\ 0 & 0 & 0 & 1 \end{array}\right]\;T_{5}=\left[\begin{array}{cccc} c_{5} & 0 & -s_{5} & a_{5}c_{5}\\ s_{5} & 0 & c_{5} & a_{5}s_{5}\\ 0 & -1 & 0 & 0\\ 0 & 0 & 0 & 1 \end{array}\right]\;T_{6}=\left[\begin{array}{cccc} c_{6} & -s_{6} & 0 & a_{6}c_{6}\\ s_{6} & c_{6} & 0 & a_{6}s_{6}\\ 0 & 0 & 1 & 0\\ 0 & 0 & 0 & 1 \end{array}\right]\\ T_{7}=\left[\begin{array}{cccc} c_{7} & -s_{7} & 0 & a_{7}c_{7}\\ s_{7} & c_{7} & 0 & a_{7}s_{7}\\ 0 & 0 & 1 & d_{7}\\ 0 & 0 & 0 & 1 \end{array}\right] \end{array}$$ (27)

$$\alpha =\mathrm{atan}2\left(\begin{array}{l} c_{7}\left(s_{6}\left(c_{5}\left(c_{2}s_{4}+c_{3}c_{4}s_{2}\right)-s_{2}s_{3}s_{5}\right)-c_{6}\left(c_{2}c_{4}-c_{3}s_{2}s_{4}\right)\right)\\ +s_{7}\left(c_{6}\left(c_{5}\left(c_{2}s_{4}+c_{3}c_{4}s_{2}\right)-s_{2}s_{3}s_{5}\right)+s_{6}\left(c_{2}c_{4}-c_{3}s_{2}s_{4}\right)\right)\\ ,\left(7s_{7}\left(s_{6}\left(c_{5}\left(c_{2}s_{4}+c_{3}c_{4}s_{2}\right)-s_{2}s_{3}s_{5}\right)-c_{6}\left(c_{2}c_{4}-c_{3}s_{2}s_{4}\right)\right)\right)/400\\ -\left(7c_{7}\left(c_{6}\left(c_{5}\left(c_{2}s_{4}+c_{3}c_{4}s_{2}\right)-s_{2}s_{3}s_{5}\right)+s_{6}\left(c_{2}c_{4}-c_{3}s_{2}s_{4}\right)\right)\right)/400\\ +\left(4999s_{5}\left(c_{2}s_{4}+c_{3}c_{4}s_{2}\right)\right)/5000\\ +\left(4999c_{5}s_{2}s_{3}\right)/5000 \end{array}\right)$$ (28)

$$\beta =a\tan2\left(\begin{array}{l} -[(4999s_{7}(s_{6}(c_{5}(c_{2}s_{4}+c_{3}c_{4}s_{2})-s_{2}s_{3}s_{5})-c_{6}(c_{2}c_{4}-c_{3}s_{2}s_{4})))/5000-(4999c_{7}(c_{6}(c_{5}(c_{2}s_{4}\\ +\;c_{3}c_{4}s_{2})-s_{2}s_{3}s_{5})+s_{6}(c_{2}c_{4}-c_{3}s_{2}s_{4})))/5000-(7s_{5}(c_{2}s_{4}+c_{3}c_{4}s_{2}))/400-(7c_{5}s_{2}s_{3})/400],sqrt([c_{7}(s_{6}\\ (c_{5}(c_{2}s_{4}+c_{3}c_{4}s_{2})-s_{2}s_{3}s_{5})-c_{6}(c_{2}c_{4}-c_{3}s_{2}s_{4}))+s_{7}(c_{6}(c_{5}(c_{2}s_{4}+c_{3}c_{4}s_{2})-s_{2}s_{3}s_{5})+s_{6}(c_{2}c_{4}-c_{3}s_{2}s_{4})){]}^{2}\\ +[(7s_{7}(s_{6}(c_{5}(c_{2}s_{4}+c_{3}c_{4}s_{2})-s_{2}s_{3}s_{5})-c_{6}(c_{2}c_{4}-c_{3}s_{2}s_{4})))/400-(7c_{7}(c_{6}(c_{5}(c_{2}s_{4}+c_{3}c_{4}s_{2})-s_{2}s_{3}s_{5})\;\\ +\;s_{6}(c_{2}c_{4}-c_{3}s_{2}s_{4})))/400+(4999s_{5}(c_{2}s_{4}+c_{3}c_{4}s_{2}))/5000+(4999c_{5}s_{2}s_{3})/5000{]}^{2}) \end{array}\right)$$ (29)

$$\eta =\mathrm{atan}2\left(\begin{array}{l} \left(7s_{5}\left(c_{4}\left(c_{1}s_{3}+c_{2}c_{3}s_{1}\right)-s_{1}s_{2}s_{4}\right)\right)/400\\ -\left(4999c_{7}\left(s_{6}\left(s_{4}\left(c_{1}s_{3}+c_{2}c_{3}s_{1}\right)+c_{4}s_{1}s_{2}\right)-c_{6}\left(c_{5}\left(c_{4}\left(c_{1}s_{3}+c_{2}c_{3}s_{1}\right)-s_{1}s_{2}s_{4}\right)+s_{5}\left(c_{1}c_{3}-c_{2}s_{1}s_{3}\right)\right)\right)\right)/5000\\ -\left(7c_{5}\left(c_{1}c_{3}-c_{2}s_{1}s_{3}\right)\right)/400\\ -\left(4999s_{7}\left(c_{6}\left(s_{4}\left(c_{1}s_{3}+c_{2}c_{3}s_{1}\right)+c_{4}s_{1}s_{2}\right)+s_{6}\left(c_{5}\left(c_{4}\left(c_{1}s_{3}+c_{2}c_{3}s_{1}\right)-s_{1}s_{2}s_{4}\right)+s_{5}\left(c_{1}c_{3}-c_{2}s_{1}s_{3}\right)\right)\right)\right)/5000\\ ,\left(4999c_{7}\left(s_{6}\left(s_{4}\left(s_{1}s_{3}-c_{1}c_{2}c_{3}\right)-c_{1}c_{4}s_{2}\right)-c_{6}\left(c_{5}\left(c_{4}\left(s_{1}s_{3}-c_{1}c_{2}c_{3}\right)+c_{1}s_{2}s_{4}\right)+s_{5}\left(s_{1}s_{3}-c_{1}c_{2}c_{3}\right)\right)\right)\right)/5000\\ -\left(7s_{5}\left(c_{4}\left(s_{1}s_{3}-c_{1}c_{2}c_{3}\right)+c_{1}s_{2}s_{4}\right)\right)/400\\ +\left(4999s_{7}\left(c_{6}\left(s_{4}\left(s_{1}s_{3}-c_{1}c_{2}c_{3}\right)-c_{1}c_{4}s_{2}\right)+s_{6}\left(c_{5}\left(c_{4}\left(s_{1}s_{3}-c_{1}c_{2}c_{3}\right)+c_{1}s_{2}s_{4}\right)+s_{5}\left(s_{1}s_{3}-c_{1}c_{2}c_{3}\right)\right)\right)\right)/5000\\ +\left(7c_{5}\left(s_{1}s_{3}-c_{1}c_{2}c_{3}\right)\right)/400 \end{array}\right)$$ (30)

$$\begin{array}{l} P_{x}=\left(\begin{array}{l} {\text{c}}_{7}\left({\text{s}}_{6}\left({\text{s}}_{4}\left({\text{s}}_{1}{\text{s}}_{3}{\;-\text{ c}}_{1}{\text{c}}_{2}{\text{c}}_{3}\right){\;-\text{ c}}_{1}{\text{c}}_{4}{\text{s}}_{2}\right){\;-\text{ c}}_{6}\left({\text{c}}_{5}\left({\text{c}}_{4}\left({\text{s}}_{1}{\text{s}}_{3}{\;-\text{ c}}_{1}{\text{c}}_{2}{\text{c}}_{3}\right){\;+\text{ c}}_{1}{\text{s}}_{2}{\text{s}}_{4}\right){\;+\text{ s}}_{5}\left({\text{c}}_{3}{\text{s}}_{1}{\;+\text{ c}}_{1}{\text{c}}_{2}{\text{s}}_{3}\right)\right)\right)\\ {+\text{ s}}_{7}\left({\text{c}}_{6}\left({\text{s}}_{4}\left({\text{s}}_{1}{\text{s}}_{3}{\;-\text{ c}}_{1}{\text{c}}_{2}{\text{c}}_{3}\right){\;-\text{ c}}_{1}{\text{c}}_{4}{\text{s}}_{2}\right){\;+\text{ s}}_{6}\left({\text{c}}_{5}\left({\text{c}}_{4}\left({\text{s}}_{1}{\text{s}}_{3}{\;-\text{ c}}_{1}{\text{c}}_{2}{\text{c}}_{3}\right){\;+\text{ c}}_{1}{\text{s}}_{2}{\text{s}}_{4}\right){\;+\text{ s}}_{5}\left({\text{c}}_{3}{\text{s}}_{1}{\;+\text{ c}}_{1}{\text{c}}_{2}{\text{s}}_{3}\right)\right)\right) \end{array}\right)\\ \left({\text{a}}_{2}{\;+\text{ a}}_{3}{\;+\text{ a}}_{4}{\;+\text{ a}}_{5}{\;+\text{ a}}_{6}{\;+\text{ a}}_{7}\right)\\ {+\text{ d}}_{7}\left(\begin{array}{l} {\text{c}}_{7}\left({\text{c}}_{6}\left({\text{s}}_{4}\left({\text{s}}_{1}{\text{s}}_{3}{\;-\text{ c}}_{1}{\text{c}}_{2}{\text{c}}_{3}\right){\;-\text{ c}}_{1}{\text{c}}_{4}{\text{s}}_{2}\right){\;+\text{ s}}_{6}\left({\text{c}}_{5}\left({\text{c}}_{4}\left({\text{s}}_{1}{\text{s}}_{3}{\;-\text{ c}}_{1}{\text{c}}_{2}{\text{c}}_{3}\right){+\text{ c}}_{1}{\text{s}}_{2}{\text{s}}_{4}\right){+\text{ s}}_{5}\left({\text{c}}_{3}{\text{s}}_{1}{\;+\text{ c}}_{1}{\text{c}}_{2}{\text{s}}_{3}\right)\right)\right)\\ {\;-\text{ s}}_{7}\left({\text{s}}_{6}\left({\text{s}}_{4}\left({\text{s}}_{1}{\text{s}}_{3}{\;-\text{ c}}_{1}{\text{c}}_{2}{\text{c}}_{3}\right){\;-\text{ c}}_{1}{\text{c}}_{4}{\text{s}}_{2}\right){\;-\text{ c}}_{6}\left({\text{c}}_{5}\left({\text{c}}_{4}\left({\text{s}}_{1}{\text{s}}_{3}{\;-\text{ c}}_{1}{\text{c}}_{2}{\text{c}}_{3}\right){+\text{ c}}_{1}{\text{s}}_{2}{\text{s}}_{4}\right){\;+\text{ s}}_{5}\left({\text{c}}_{3}{\text{s}}_{1}{\;+\text{ c}}_{1}{\text{c}}_{2}{\text{s}}_{3}\right)\right)\right) \end{array}\right)\\ {\;+\text{ d}}_{1}\left({\text{s}}_{5}\left({\text{c}}_{4}\left({\text{s}}_{1}{\text{s}}_{3}{\;-\text{ c}}_{1}{\text{c}}_{2}{\text{c}}_{3}\right){\;+\text{ c}}_{1}{\text{s}}_{2}{\text{s}}_{4}\right){\;-\text{ c}}_{5}\left({\text{c}}_{3}{\text{s}}_{1}{\;+\text{ c}}_{1}{\text{c}}_{2}{\text{s}}_{3}\right)\right)\\ {+\text{ d}}_{7}\left({\text{s}}_{5}\left({\text{c}}_{4}\left({\text{s}}_{1}{\text{s}}_{3}{\;-\text{ c}}_{1}{\text{c}}_{2}{\text{c}}_{3}\right){\;+\text{ c}}_{1}{\text{s}}_{2}{\text{s}}_{4}\right){\;-\text{ c}}_{5}\left({\text{c}}_{3}{\text{s}}_{1}{\;+\text{ c}}_{1}{\text{c}}_{2}{\text{s}}_{3}\right)\right){\;-\text{ a}}_{5}{\text{c}}_{5}\left({\text{c}}_{4}\left({\text{s}}_{1}{\text{s}}_{3}{\;-\text{ c}}_{1}{\text{c}}_{2}{\text{c}}_{3}\right){\;+\text{ c}}_{1}{\text{s}}_{2}{\text{s}}_{4}\right)\\ {+\text{ a}}_{7}{\text{c}}_{7}\left({\text{s}}_{6}\left({\text{s}}_{4}\left({\text{s}}_{1}{\text{s}}_{3}{\;-\text{ c}}_{1}{\text{c}}_{2}{\text{c}}_{3}\right){\;-\text{ c}}_{1}{\text{c}}_{4}{\text{s}}_{2}\right){\;-\text{ c}}_{6}\left({\text{c}}_{5}\left({\text{c}}_{4}\left({\text{s}}_{1}{\text{s}}_{3}{\;-\text{ c}}_{1}{\text{c}}_{2}{\text{c}}_{3}\right){\;+\text{ c}}_{1}{\text{s}}_{2}{\text{s}}_{4}\right){\;+\text{ s}}_{5}\left({\text{c}}_{3}{\text{s}}_{1}{\;+\text{ c}}_{1}{\text{c}}_{2}{\text{s}}_{3}\right)\right)\right)\\ {+\text{ a}}_{6}{\text{s}}_{6}\left({\text{s}}_{4}\left({\text{s}}_{1}{\text{s}}_{3}{\;-\text{ c}}_{1}{\text{c}}_{2}{\text{c}}_{3}\right){\;-\text{ c}}_{1}{\text{c}}_{4}{\text{s}}_{2}\right)\\ {+\text{ a}}_{7}{\text{s}}_{7}\left({\text{c}}_{6}\left({\text{s}}_{4}\left({\text{s}}_{1}{\text{s}}_{3}{\;-\text{ c}}_{1}{\text{c}}_{2}{\text{c}}_{3}\right){\;-\text{ c}}_{1}{\text{c}}_{4}{\text{s}}_{2}\right){\;+\text{ s}}_{6}\left({\text{c}}_{5}\left({\text{c}}_{4}\left({\text{s}}_{1}{\text{s}}_{3}{\;-\text{ c}}_{1}{\text{c}}_{2}{\text{c}}_{3}\right){+\text{ c}}_{1}{\text{s}}_{2}{\text{s}}_{4}\right){\;+\text{ s}}_{5}\left({\text{c}}_{3}{\text{s}}_{1}{\;+\text{ c}}_{1}{\text{c}}_{2}{\text{s}}_{3}\right)\right)\right)\\ {-\text{ a}}_{6}{\text{c}}_{6}\left({\text{c}}_{5}\left({\text{c}}_{4}\left({\text{s}}_{1}{\text{s}}_{3}{\;-\text{ c}}_{1}{\text{c}}_{2}{\text{c}}_{3}\right){\;+\text{ c}}_{1}{\text{s}}_{2}{\text{s}}_{4}\right){\;+\text{ s}}_{5}\left({\text{c}}_{3}{\text{s}}_{1}{\;+\text{ c}}_{1}{\text{c}}_{2}{\text{s}}_{3}\right)\right)\\ {-\text{ a}}_{4}{\text{c}}_{4}\left({\text{s}}_{1}{\text{s}}_{3}{\;-\text{ c}}_{1}{\text{c}}_{2}{\text{c}}_{3}\right){\;-\text{ a}}_{5}{\text{s}}_{5}\left({\text{c}}_{3}{\text{s}}_{1}{\;+\text{ c}}_{1}{\text{c}}_{2}{\text{s}}_{3}\right){\;+\text{ a}}_{2}{\text{c}}_{1}{\text{c}}_{2}{\;-\text{ a}}_{3}{\text{s}}_{1}{\text{s}}_{3}{\;-\text{ a}}_{4}{\text{c}}_{1}{\text{s}}_{2}{\text{s}}_{4}{\;+\text{ a}}_{3}{\text{c}}_{1}{\text{c}}_{2}{\text{c}}_{3} \end{array}$$ (31)

$$\begin{array}{l} P_{\text{y}}={\text{a}}_{5}{\text{c}}_{5}\left({\text{c}}_{4}\left({\text{c}}_{1}{\text{s}}_{3}{\;+\text{ c}}_{2}{\text{c}}_{3}{\text{s}}_{1}\right){\;-\text{ s}}_{1}{\text{s}}_{2}{\text{s}}_{4}\right)\\ -\;\left(\begin{array}{l} {\text{c}}_{7}\left({\text{s}}_{6}\left({\text{s}}_{4}\left({\text{c}}_{1}{\text{s}}_{3}{\;+\text{ c}}_{2}{\text{c}}_{3}{\text{s}}_{1}\right){\;+\text{ c}}_{4}{\text{s}}_{1}{\text{s}}_{2}\right){\;-\text{ c}}_{6}\left({\text{c}}_{5}\left({\text{c}}_{4}\left({\text{c}}_{1}{\text{s}}_{3}{\;+\text{ c}}_{2}{\text{c}}_{3}{\text{s}}_{1}\right){-\text{ s}}_{1}{\text{s}}_{2}{\text{s}}_{4}\right){\;+\text{ s}}_{5}\left({\text{c}}_{1}{\text{c}}_{3}{\;-\text{ c}}_{2}{\text{s}}_{1}{\text{s}}_{3}\right)\right)\right)\\ {\;+\text{ s}}_{7}\left({\text{c}}_{6}\left({\text{s}}_{4}\left({\text{c}}_{1}{\text{s}}_{3}{\;+\text{ c}}_{2}{\text{c}}_{3}{\text{s}}_{1}\right){\;+\text{ c}}_{4}{\text{s}}_{1}{\text{s}}_{2}\right){\;+\text{ s}}_{6}\left({\text{c}}_{5}\left({\text{c}}_{4}\left({\text{c}}_{1}{\text{s}}_{3}{\;+\text{ c}}_{2}{\text{c}}_{3}{\text{s}}_{1}\right){\;-\text{ s}}_{1}{\text{s}}_{2}{\text{s}}_{4}\right){\;+\text{ s}}_{5}\left({\text{c}}_{1}{\text{c}}_{3}{\;-\text{ c}}_{2}{\text{s}}_{1}{\text{s}}_{3}\right)\right)\right) \end{array}\right)\\ \left({\text{a}}_{2}{\;+\text{ a}}_{3}{\;+\text{ a}}_{4}{\;+\text{ a}}_{5}{\;+\text{ a}}_{6}{\;+\text{ a}}_{7}\right){\;-\text{ d}}_{1}\left({\text{s}}_{5}\left({\text{c}}_{4}\left({\text{c}}_{1}{\text{s}}_{3}{\;+\text{ c}}_{2}{\text{c}}_{3}{\text{s}}_{1}\right){\;-\text{ s}}_{1}{\text{s}}_{2}{\text{s}}_{4}\right){\;-\text{ c}}_{5}\left({\text{c}}_{1}{\text{c}}_{3}{\;-\text{ c}}_{2}{\text{s}}_{1}{\text{s}}_{3}\right)\right)\\ {-\text{ d}}_{7}\left({\text{s}}_{5}\left({\text{c}}_{4}\left({\text{c}}_{1}{\text{s}}_{3}{\;+\text{ c}}_{2}{\text{c}}_{3}{\text{s}}_{1}\right){\;-\text{ s}}_{1}{\text{s}}_{2}{\text{s}}_{4}\right){\;-\text{ c}}_{5}\left({\text{c}}_{1}{\text{c}}_{3}{\;-\text{ c}}_{2}{\text{s}}_{1}{\text{s}}_{3}\right)\right)\\ {-\text{ d}}_{7}\left(\begin{array}{l} {\text{c}}_{7}\left({\text{c}}_{6}\left({\text{s}}_{4}\left({\text{c}}_{1}{\text{s}}_{3}{\;+\text{ c}}_{2}{\text{c}}_{3}{\text{s}}_{1}\right){\;+\text{ c}}_{4}{\text{s}}_{1}{\text{s}}_{2}\right){+\text{ s}}_{6}\left({\text{c}}_{5}\left({\text{c}}_{4}\left({\text{c}}_{1}{\text{s}}_{3}{\;+\text{ c}}_{2}{\text{c}}_{3}{\text{s}}_{1}\right){\;-\text{ s}}_{1}{\text{s}}_{2}{\text{s}}_{4}\right){\;+\text{ s}}_{5}\left({\text{c}}_{1}{\text{c}}_{3}{\;-\text{ c}}_{2}{\text{s}}_{1}{\text{s}}_{3}\right)\right)\right)\\ {\;-\text{ s}}_{7}\left({\text{s}}_{6}\left({\text{s}}_{4}\left({\text{c}}_{1}{\text{s}}_{3}{\;+\text{ c}}_{2}{\text{c}}_{3}{\text{s}}_{1}\right){+\text{ c}}_{4}{\text{s}}_{1}{\text{s}}_{2}\right){\;-\text{ c}}_{6}\left({\text{c}}_{5}\left({\text{c}}_{4}\left({\text{c}}_{1}{\text{s}}_{3}{\;+\text{ c}}_{2}{\text{c}}_{3}{\text{s}}_{1}\right){\;-\text{ s}}_{1}{\text{s}}_{2}{\text{s}}_{4}\right){+\text{ s}}_{5}\left({\text{c}}_{1}{\text{c}}_{3}{\;-\text{ c}}_{2}{\text{s}}_{1}{\text{s}}_{3}\right)\right)\right) \end{array}\right)\\ {-\text{ a}}_{6}{\text{s}}_{6}\left({\text{s}}_{4}\left({\text{c}}_{1}{\text{s}}_{3}{\;+\text{ c}}_{2}{\text{c}}_{3}{\text{s}}_{1}\right){\;+\text{ c}}_{4}{\text{s}}_{1}{\text{s}}_{2}\right)\;\\ {-\text{ a}}_{7}{\text{c}}_{7}\left({\text{s}}_{6}\left({\text{s}}_{4}\left({\text{c}}_{1}{\text{s}}_{3}{\;+\text{ c}}_{2}{\text{c}}_{3}{\text{s}}_{1}\right){+\text{ c}}_{4}{\text{s}}_{1}{\text{s}}_{2}\right){\;-\text{ c}}_{6}\left({\text{c}}_{5}\left({\text{c}}_{4}\left({\text{c}}_{1}{\text{s}}_{3}{\;+\text{ c}}_{2}{\text{c}}_{3}{\text{s}}_{1}\right){\;-\text{ s}}_{1}{\text{s}}_{2}{\text{s}}_{4}\right){+\text{ s}}_{5}\left({\text{c}}_{1}{\text{c}}_{3}{\;-\text{ c}}_{2}{\text{s}}_{1}{\text{s}}_{3}\right)\right)\right)\\ {+\text{ a}}_{6}{\text{c}}_{6}\left({\text{c}}_{5}\left({\text{c}}_{4}\left({\text{c}}_{1}{\text{s}}_{3}{\;+\text{ c}}_{2}{\text{c}}_{3}{\text{s}}_{1}\right){\;-\text{ s}}_{1}{\text{s}}_{2}{\text{s}}_{4}\right){+\text{ s}}_{5}\left({\text{c}}_{1}{\text{c}}_{3}{\;-\text{ c}}_{2}{\text{s}}_{1}{\text{s}}_{3}\right)\right)\\ {+\text{ a}}_{4}{\text{c}}_{4}\left({\text{c}}_{1}{\text{s}}_{3}{\;+\text{ c}}_{2}{\text{c}}_{3}{\text{s}}_{1}\right){-\text{ a}}_{7}{\text{s}}_{7}\left({\text{c}}_{6}\left({\text{s}}_{4}\left({\text{c}}_{1}{\text{s}}_{3}{\;+\text{ c}}_{2}{\text{c}}_{3}{\text{s}}_{1}\right){\;+\text{ c}}_{4}{\text{s}}_{1}{\text{s}}_{2}\right){+\text{ s}}_{6}\left({\text{c}}_{5}\left({\text{c}}_{4}\left({\text{c}}_{1}{\text{s}}_{3}{\;+\text{ c}}_{2}{\text{c}}_{3}{\text{s}}_{1}\right){-\text{ s}}_{1}{\text{s}}_{2}{\text{s}}_{4}\right){\;+\text{ s}}_{5}\left({\text{c}}_{1}{\text{c}}_{3}{\;-\text{ c}}_{2}{\text{s}}_{1}{\text{s}}_{3}\right)\right)\right)\\ {+\text{ a}}_{5}{\text{s}}_{5}\left({\text{c}}_{1}{\text{c}}_{3}{\;-\text{ c}}_{2}{\text{s}}_{1}{\text{s}}_{3}\right){+\text{ a}}_{2}{\text{c}}_{2}{\text{s}}_{1}{\;+\text{ a}}_{3}{\text{c}}_{1}{\text{s}}_{3}{\;-\text{ a}}_{4}{\text{s}}_{1}{\text{s}}_{2}{\text{s}}_{4}{\;+\text{ a}}_{3}{\text{c}}_{2}{\text{c}}_{3}{\text{s}}_{1} \end{array}$$ (32)

$$\begin{array}{l} P_{z}={\text{d}}_{1}{\;+\text{ d}}_{7}\left(\begin{array}{l} {\text{c}}_{7}\left({\text{s}}_{6}\left({\text{c}}_{5}\left({\text{c}}_{2}{\text{s}}_{4}{\;+\text{ c}}_{3}{\text{c}}_{4}{\text{s}}_{2}\right){-\text{ s}}_{2}{\text{s}}_{3}{\text{s}}_{5}\right){\;-\text{ c}}_{6}\left({\text{c}}_{2}{\text{c}}_{4}{\;-\text{ c}}_{3}{\text{s}}_{2}{\text{s}}_{4}\right)\right)\\ {+\text{ s}}_{7}\left({\text{c}}_{6}\left({\text{c}}_{5}\left({\text{c}}_{2}{\text{s}}_{4}{\;+\text{ c}}_{3}{\text{c}}_{4}{\text{s}}_{2}\right){-\text{ s}}_{2}{\text{s}}_{3}{\text{s}}_{5}\right){+\text{ s}}_{6}\left({\text{c}}_{2}{\text{c}}_{4}{\;-\text{ c}}_{3}{\text{s}}_{2}{\text{s}}_{4}\right)\right) \end{array}\right)\\ {-\text{ a}}_{2}{\text{s}}_{2}\;-\;\left({\text{c}}_{7}\left({\text{c}}_{6}\left({\text{c}}_{5}\left({\text{c}}_{2}{\text{s}}_{4}{\;+\text{ c}}_{3}{\text{c}}_{4}{\text{s}}_{2}\right){\;-\text{ s}}_{2}{\text{s}}_{3}{\text{s}}_{5}\right){+\text{ s}}_{6}\left({\text{c}}_{2}{\text{c}}_{4}{\;-\text{ c}}_{3}{\text{s}}_{2}{\text{s}}_{4}\right)\right)\right)\\ {\;-\text{ s}}_{7}\left({\text{s}}_{6}\left({\text{c}}_{5}\left({\text{c}}_{2}{\text{s}}_{4}{+\text{ c}}_{3}{\text{c}}_{4}{\text{s}}_{2}\right){-\text{ s}}_{2}{\text{s}}_{3}{\text{s}}_{5}\right){\;-\text{ c}}_{6}\left({\text{c}}_{2}{\text{c}}_{4}{\;-\text{ c}}_{3}{\text{s}}_{2}{\text{s}}_{4}\right)\right)\\ \left({\text{a}}_{2}{\;+\text{ a}}_{3}{\;+\text{ a}}_{4}{\;+\text{ a}}_{5}{\;+\text{ a}}_{6}{\;+\text{ a}}_{7}\right){\;+\text{ d}}_{1}\left({\text{s}}_{5}\left({\text{c}}_{2}{\text{s}}_{4}{\;+\text{ c}}_{3}{\text{c}}_{4}{\text{s}}_{2}\right){+\text{ c}}_{5}{\text{s}}_{2}{\text{s}}_{3}\right)\\ {+\text{ d}}_{7}\left({\text{s}}_{5}\left({\text{c}}_{2}{\text{s}}_{4}{\;+\text{ c}}_{3}{\text{c}}_{4}{\text{s}}_{2}\right){+\text{ c}}_{5}{\text{s}}_{2}{\text{s}}_{3}\right){\;-\text{ a}}_{6}{\text{c}}_{6}\left({\text{c}}_{5}\left({\text{c}}_{2}{\text{s}}_{4}{\;+\text{ c}}_{3}{\text{c}}_{4}{\text{s}}_{2}\right){\;-\text{ s}}_{2}{\text{s}}_{3}{\text{s}}_{5}\right)\\ {\;-\text{ a}}_{7}{\text{c}}_{7}\left({\text{c}}_{6}\left({\text{c}}_{5}\left({\text{c}}_{2}{\text{s}}_{4}{\;+\text{ c}}_{3}{\text{c}}_{4}{\text{s}}_{2}\right){-\text{ s}}_{2}{\text{s}}_{3}{\text{s}}_{5}\right){+\text{ s}}_{6}\left({\text{c}}_{2}{\text{c}}_{4}{\;-\text{ c}}_{3}{\text{s}}_{2}{\text{s}}_{4}\right)\right)\\ {-\text{ a}}_{5}{\text{c}}_{5}\left({\text{c}}_{2}{\text{s}}_{4}{\;+\text{ c}}_{3}{\text{c}}_{4}{\text{s}}_{2}\right){+\text{ a}}_{7}{\text{s}}_{7}\left({\text{s}}_{6}\left({\text{c}}_{5}\left({\text{c}}_{2}{\text{s}}_{4}{\;+\text{ c}}_{3}{\text{c}}_{4}{\text{s}}_{2}\right){-\text{ s}}_{2}{\text{s}}_{3}{\text{s}}_{5}\right){\;-\text{ c}}_{6}\left({\text{c}}_{2}{\text{c}}_{4}{\;-\text{ c}}_{3}{\text{s}}_{2}{\text{s}}_{4}\right)\right)\\ {-\text{ a}}_{6}{\text{s}}_{6}\left({\text{c}}_{2}{\text{c}}_{4}{\;-\text{ c}}_{3}{\text{s}}_{2}{\text{s}}_{4}\right){\;-\text{ a}}_{3}{\text{c}}_{3}{\text{s}}_{2}{\;-\text{ a}}_{4}{\text{c}}_{2}{\text{s}}_{4}{\;+\text{ a}}_{5}{\text{s}}_{2}{\text{s}}_{3}{\text{s}}_{5}{\;-\text{ a}}_{4}{\text{c}}_{3}{\text{c}}_{4}{\text{s}}_{2} \end{array}$$ (33)

where $s_{i}=\sin\theta_{i}\left(i=1∼6\right)$ , $c_{i}=\cos\theta_{i}\left(i=1∼6\right)$ . $a_{1}∼a_{7},d_{1}∼d_{7}$ denotes the parameters of Seven-degree-of-freedom robot arm. g represents the initial position attitude matrix of the end-effector, $T_{1}∼T_{7}$ represents the variation matrix of the joint angles from 1 to 6, α,β,η represents Euler angles of rotation along the *x*,*y*,*z* axes after the end-effector moves from the initial position, and $P_{x},P_{y},P_{z}$ represents the new position of the end-effector after the end-effector moves from the initial position.

## 4 Particle Swarm Algorithm and Enhancement

### 4.1 Two Particle Swarm Algorithms

To solve practical engineering applications, researchers have invented metaheuristic algorithms based on the laws of nature. Particle swarm optimization algorithm, as a kind of metaheuristic algorithm, is an algorithm invented to simulate bird flock predation. Based on the feature that the flock of birds close to the predation target will drive the flock of birds at a distance to the predation target and eventually drive the flock as a whole to the predation target, the particles of the population in the particle swarm algorithm will carry two variables, x and ν . Meanwhile, the solution will be approximated to the optimal solution by updating x and ν in real time. Where x denotes the position of the particle in the search space and ν denotes the step length and direction of movement. For the classical particle swarm algorithm, the update formula for ν and x is shown below.

$$\nu_{i}^{t+1}=\omega \nu_{i}^{t}+c_{1}r_{1}\left(P_{\text{best}}-x_{i}^{t}\right)+c_{2}r_{2}\left(G_{best}-x_{i}^{t}\right)$$ (34)

$$x_{i}^{t+1}=x_{i}^{t}+\nu_{i}^{t+1}$$ (35)

Where i represents the position of the particle in the population, t represents the number of iterations, and ω represents the inertia weight. $c_{1}$ represents the cognitive learning factor, and $c_{2}$ represents the social learning factor, both of which are generally taken between 1 and 2. $r_{1}$ and $r_{2}$ are random numbers between 0 and 1. $P_{\text{best}}$ represents the individual optimal position, and $G_{best}$ represents the global optimal position. The above is the update formula of position as well as velocity of the classical particle swarm algorithm. In addition to the above update formula, there is a quantum mechanics-based position update method in the QPSO algorithm, which discards the velocity update and chooses the position update method, which is a novel attempt, and its update formula is shown below.

$$g_{i}=\varphi pbest_{i}+\left(1-\varphi \right)gbest_{d}$$ (36)

$$x_{i}\left(t+1\right)=g_{i}+\beta \left|mbest_{d}-x_{i}\left(t\right)\right|\log\left(\frac{1}{u}\right)\;u\text{>0}\text{.}5$$ (37)

$$x_{i}\left(t+1\right)=g_{i}-\beta \left|mbest_{d}-x_{i}\left(t\right)\right|\log\left(\frac{1}{u}\right)\;u\text{>0}\text{.}5$$ (38)

$$mbest_{d}=\frac{1}{M}\sum_{i=1}^{M}pbest_{i}$$ (39)

where i represents the position of the particle in the population, t represents the number of iterations, and M represents the number of populations. φ , u is a random number between 0 and 1, and β is a constant number between 0 and 1. The pbest represents the best position of the individual particle, gbest represents the optimal position of the population, and mbest represents the average of the best position of the individual particle. The basic steps of solving the particle swarm algorithm are shown below.

Step 1: Initialization of the population particles.

Step 2: Calculate the fitness function.

Step 3: Update particle position and velocity.

Step 4: Update individual best position and group best position.

### 4.2 Improvement

For setting the weights when updating the velocity in the particle swarm algorithm, this paper adopts the adaptive weights. The most important feature of the adaptive weights is that the weights change with the change of the fitness function value of the particles. For different weights, the local search as well as the global search capability of the algorithm will be very different. Generally large values of inertia weights are more favorable for global search, and small values of inertia weights are favorable for local search. When the algorithm falls into the local optimum, it tends to miss the optimal solution and then converge too early, while when the global search ability of the algorithm is too strong, the final accuracy of the algorithm is often not too high. In order to balance the local search as well as the global search ability, adaptive weights are the best choice. The basic idea is as follows:

Step 1: Calculate value of fitness function fitness , minimum fitness function value $fitness_{\min}$ and average fitness function value $fitness_{favg}$ .

Step 2: If fitness is less than or equal to $fitness_{favg}$ , the weight become:

$$\omega =\omega_{\min}+\frac{\left(\omega_{\max}-\omega_{\min}\right)\left(fitness-fitness_{\min}\right)}{\left(fitness_{favg}-fitness_{\min}\right)}$$ (40)

If the fitness is larger than fitness , the weight become: $\omega =\omega_{\max}$ .

Step 3: Update fitness .

Particle swarm algorithms require random initialization of positions and velocities, and there is no fixed standard for initialization, which makes it difficult to guarantee the accuracy and precision of the final solution, and the search time is often too long, resulting in low efficiency of the algorithm. Most researchers use particle swarm algorithms to study the robot inverse kinematics solution without further description of the initialization process of the population, but only add boundary conditions to the algorithm to ensure the executability of the algorithm. Based on the consideration of the shortest algorithm operation time, this paper adopts the conditional restriction based on the limit joints, and the maximum as well as the minimum values of the position are determined by the range of values of each joint angle. For the maximum and minimum values of velocity, this paper introduces the position coefficient k and adopts the form of multiplying position and coefficient to determine the velocity factor, and the specific conditions are set as follows.

Step 1: Determine maximum and minimum position $x_{\max}$ , $x_{\min}$ according to the range of values of the joint angle.

Step 2: Determine maximum and minimum speed $\nu_{\max}$ , $\nu_{\min}$ according to $x_{\max}$ , $x_{\min}$ , where:

$$\nu_{\max}=x_{\max}\cdot k;\;\nu_{\min}=x_{\max}\cdot \left(-k\right)$$

(Based on actual experience k takes the value of 0.5)

Step 3: Determine position x and speed ν .

$$x=\text{rand}\left(0,1\right)\left(x_{\max}-x_{\min}\right)+x_{\min}$$ (41)

$$\nu =\text{rand}\left(0,1\right)\left(\nu_{\max}-\nu_{\min}\right)+\nu_{\min}$$ (42)

## 5 Inverse Kinematic Solution Using Improved Particle Swarm Algorithm

### 5.1 Fitness Function

**FIGURE 2:**
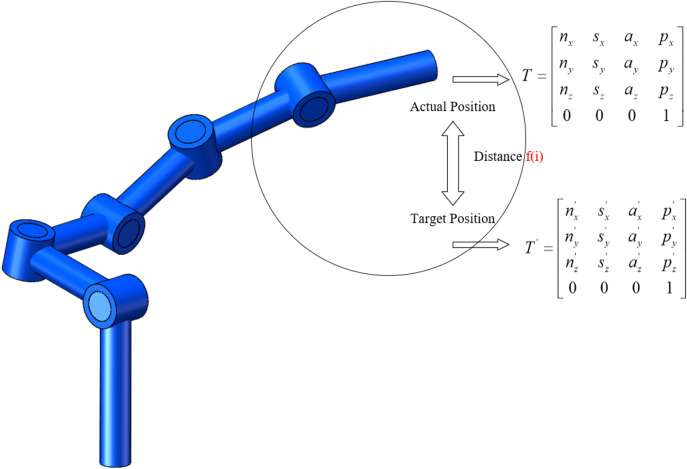
Schematic diagram of the fitness function.

The selection of the fitness function greatly affects the efficiency of the particle swarm algorithm. For the robot arm inverse kinematics solution problem, in order to better ensure the accuracy of position and direction solution, the form of the fitness function in this paper is the error value of the robot arm end-effector position and direction angle. The error is selected as the Euclidean distance between the target position, direction angle and the actual position and direction angle. The specific form of the fitness function is shown in Eq. 43. A geometric illustration of the fitness function is shown in Figure 2.

$$f\left(i\right)=\sqrt{\sum_{i=1}^{3}{\left(T_{i1}^{'}-T_{i1}\right)}^{2}+\sum_{i=1}^{3}{\left(T_{i2}^{'}-T_{i2}\right)}^{2}+\sum_{i=1}^{3}{\left(T_{i3}^{'}-T_{i3}\right)}^{2}+\sum_{i=1}^{3}{\left(T_{i4}^{'}-T_{i4}\right)}^{2}}$$ (43)

In Eq. 43, the first to third terms under the root sign represent the directional angle error of the deflection of the robotic arm end-effector along the positive direction of the x, y, and *z* axes, respectively, and the fourth term under the root sign represents the position error of the robotic arm end-effector. In Figure 2, the geometric meaning of the adaptation function is further illustrated. When the robotic arm end-effector cannot reach the specified target position, the Euclidean distance between the actual position and attitude of the robotic arm end-effector and the ideal position and attitude is represented as the error, and this error is reflected in the form of the fitness function. Eq. 43 and the matrix T in Figure 2 represent the actual end position pose matrix of the robotic arm end-effector. t' represents the ideal end position pose matrix of the robotic arm end-effector. Where the matrix forms of T and T′ are shown in Eqs. 44 and 45, respectively.

$$T=\left[\begin{array}{cccc} n_{x} & s_{x} & a_{x} & p_{x}\\ n_{y} & s_{y} & a_{y} & p_{y}\\ n_{z} & s_{z} & a_{z} & p_{z}\\ 0 & 0 & 0 & 1 \end{array}\right]$$ (44)

$$T^{'}=\left[\begin{array}{cccc} n_{x}^{'} & s_{x}^{'} & a_{x}^{'} & p_{x}^{'}\\ n_{y}^{'} & s_{y}^{'} & a_{y}^{'} & p_{y}^{'}\\ n_{z}^{'} & s_{z}^{'} & a_{z}^{'} & p_{z}^{'}\\ 0 & 0 & 0 & 1 \end{array}\right]$$ (45)

In Eq. 44, the first three rows and the first three columns of the matrix T form the rotation transformation matrix, which represents the actual attitude transformation of the end-effector of the robot arm, and the first three rows of the last column of the matrix T form the column vector, which represents the actual position of the end-effector of the robot arm with respect to the base coordinate system. In Eq. 45, the first three rows and the first three columns of the matrix T′ form the rotation transformation matrix, which represents the target attitude transformation of the end-effector of the robot arm, and the first three rows of the last column of the matrix T′ form the column vector, which represents the target position to be reached by the end-effector of the robot arm.

### 5.2 Flowchart and Pseudocode

**FIGURE 3:**
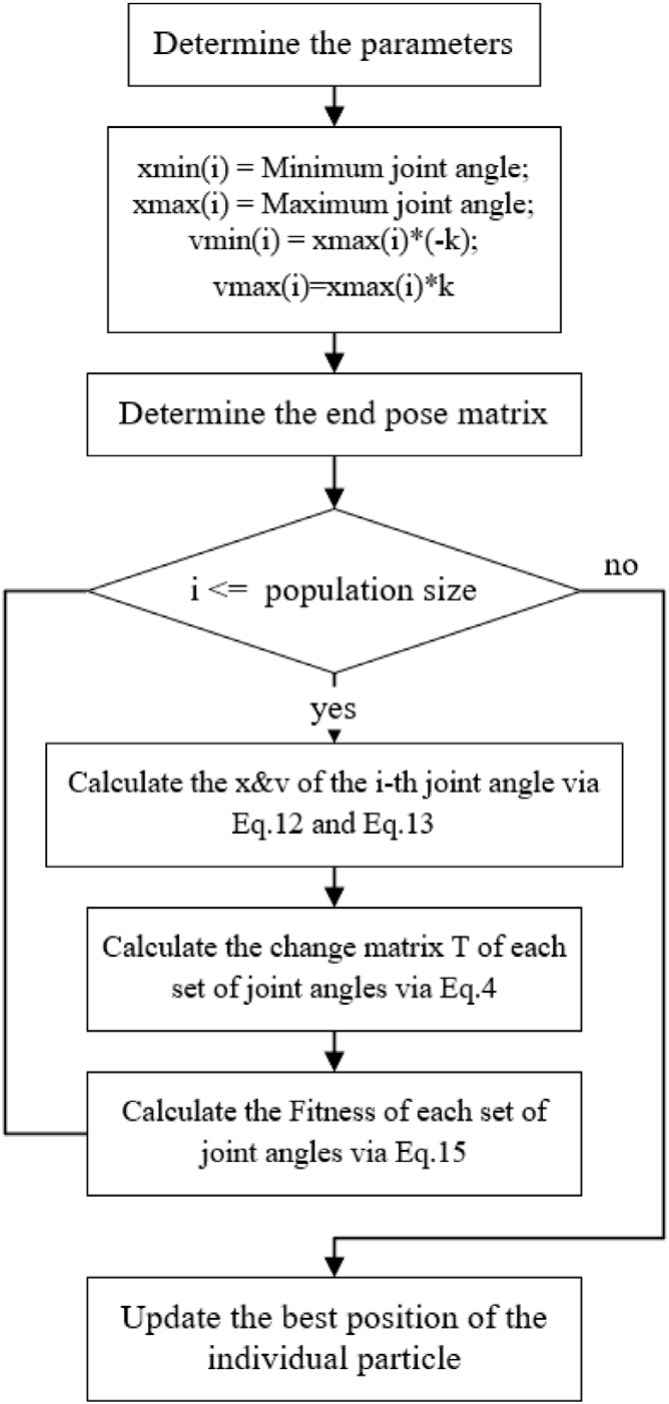
Intialize process.

**FIGURE 4:**
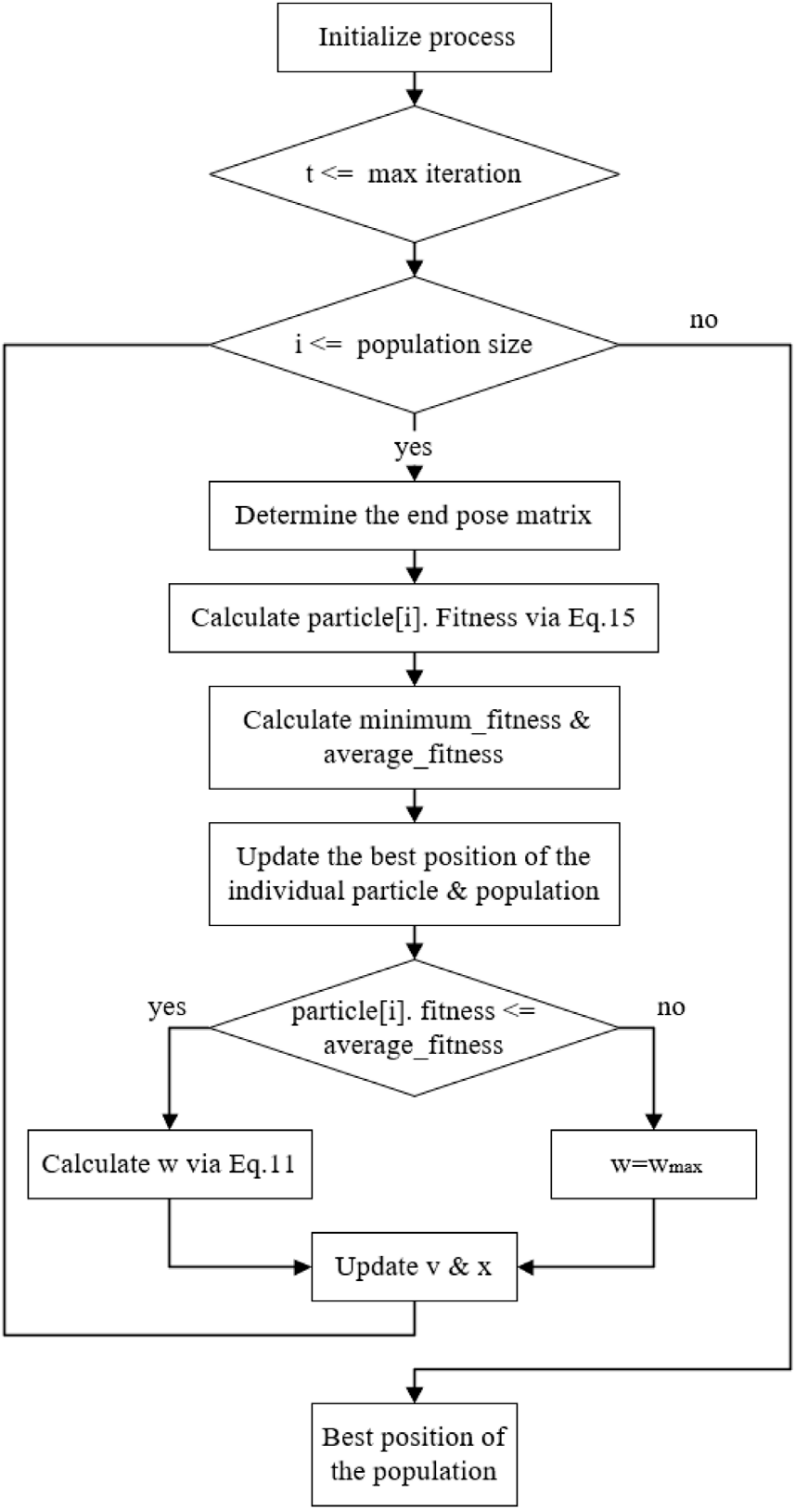
Main loop.

The flowchart and the pseudo-code of the improved PSO algorithm proposed in this paper are shown in Figures 3, 4, and Table 1, respectively. Where Fgure 3 represents the parameter initialization process based on limit joints. Figure 4 shows the main iterative loop process for updating the position as well as the velocity.

**TABLE 1:** Values of relevant parameters of general industrial six-degree-of-freedom robotic arms and the range of values of each joint angle.

d_1_(m)	a_1_(m)	a_2_(m)	a_3_(m)	d_4_(m)	a_6_(m)	d_6_(m)	Range of $\theta_{1}(\text{°})$ (i = 1–6)
0.25	0.15	0.55	0.16	0.594	0.1	0.1	(-π, π]


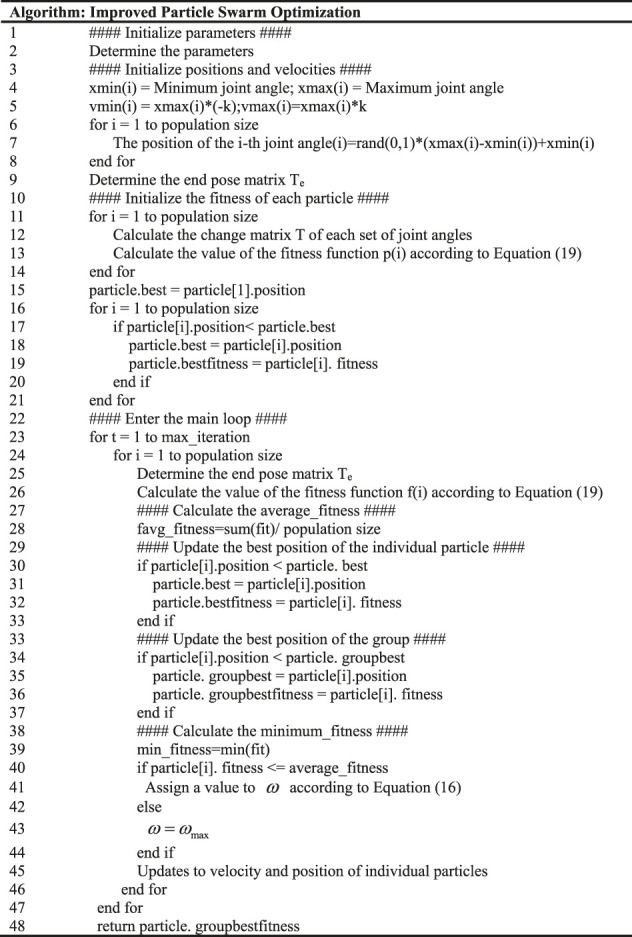


## 6 Experiments and Results

There are three experiments in this paper. The first experiment is an inverse kinematic solution for a general six-degree-of-freedom industrial robotic arm using an improved particle swarm algorithm based on the spinor modeling method. The second experiment compares different improved particle swarm algorithms in terms of algorithm accuracy, convergence and operation time based on existing references for PUMA560 robotic arm. The third experiment replaces the object of study with a seven-degree-of-freedom robotic arm and repeats the steps of experiment 2. The criterion for comparing the algorithms in experiment 2 and experiment 3 was to set the same number of iterations, with the same initial parameters. The criteria for evaluating the algorithm’s capability include the comparison of solution accuracy, solution time, and generalizability. The experiments were all coded in MATLAB R2021b with the processor model: Intel (R) Core (TM) i9-12900KF CPU @ 3.19GHz.

### 6.1 Results Obtained for General Industrial Six-degree-of-freedom Robotic Arm

The general industrial six-degree-of-freedom robotic arm is studied above, and its positive kinematic model is established based on the rotating body theory. In Experiment 1, the relevant parameter values and the ranges of each joint angle of the general industrial six-degree-of-freedom robotic arm are given in Table 1. And the initial position of the end-effector of the robotic arm and the Euler angles of rotation along *x*, *y*, *z* axes are set, as shown in Table 2. Also, the actual impact points of the four sets of robotic arm end-effectors are set, and the positions of the end-effectors at the points and the Euler angles of rotation along the *x*, *y*, *z* axes are given in Table 3. The robotic arm end-effector moves from the initial position to the impact point in the process of the robotic arm realizes the change from one position attitude to another position attitude.

**TABLE 2:** Initial position of the end-effector of the robot arm and the orientation angle.

Initial Position of the End-Effector			Initial Azimuth of the End-Effector		
${\text{x}}_{\text{0}}\left(m\right)$	${\text{y}}_{\text{0}}\left(m\right)$	${\text{z}}_{\text{0}}\left(m\right)$	$\alpha_{\text{0}}\left(rad\right)$	$\beta_{\text{0}}\left(rad\right)$	$\eta_{\text{0}}\left(rad\right)$
0	a_1_+d_4_+a_6_	d_1_+a_2_+a_3_-d_6_	0	0	0

**TABLE 3:** Position and orientation angle of the end-effector corresponding to the given impact point.

N°	Position Error of the End-Effector			Euler Angles Error of the End-Effector		
	x0(m)E	y0(m)E	x0(m)E	α0(rad)E	β0(rad)E	η0(rad)E
1	0.1,154,947	0.2,725,154	0.256,376	−2.817,121	0.6,356,652	0.9,973,017
2	−0.0009,583,057	0.2,715,589	0.1,660,651	−2.886,616	0.3,067,386	1.770,458
3	−0.1,268,636	0.3,495,127	0.1,782,351	−2.708,327	0.02,164,443	2.482,109
4	−0.1,676,911	0.4,555,857	0.2,694,087	−2.387,233	−0.07,313,938	3.231,621

Tables 1–3 show the conditions. $a_{1},a_{2},a_{3},a_{6},d_{1},d_{4},d_{6}$ in Table 1 indicates the parameters of a general six-degree-of-freedom industrial robotic arm, and $\theta_{i}$ indicates the joint angle i (i = 1–6). In Table 2, ${\text{x}}_{\text{0}},{\text{y}}_{\text{0}},{\text{z}}_{0}$ denotes the initial position of the robotic arm end-effector, $\alpha_{0},\beta_{0},\eta_{0}$ denotes the initial Euler angles of the robotic arm end-effector rotated along the x, y, and *z* axes, respectively. In Table 3, XE0,YE0,ZE0 denotes the new position of the robotic arm end-effector after moving from the initial position, and αE0,βE0,ηE0 denotes the Euler angles of the robotic arm end-effector rotating along the *x*, *y*, *z* axes after moving from the initial position, respectively. The initial position of the end-effector of the robot arm and the Euler angles of rotation along the *x*,*y*,*z* axes set in Table 2 are known, and the position of the end-effector of the robot arm after moving and the Euler angles of rotation along the *x*, *y*, *z* axes can be obtained by combining the positive kinematic Eq. 9. According to Table 3, the specific position of the end-effector of the robot arm after moving and the Euler angles of rotation along *x*, *y*, *z* axes can be obtained. The error expression, i.e., the fitness function, is obtained by converting the two previous parts into a matrix and making a difference. By solving the minimum value of the fitness function, the algorithm finally obtains the joint angle corresponding to the smallest error and the specific position of the robot arm end-effector after moving and the Euler angle of rotation along the *x*, *y*, *z* axes. The algorithm can find out the six joint angles corresponding to the end-effector impact point out after the robot arm moves, as shown in Table 4. By comparing the actual position of the end-effector and the Euler angles of rotation along the *x*, *y*, *z* axes with the position of the given point and the Euler angles of rotation along the *x*,*y*,*z* axes, it can be shown to some extent that the algorithm can guarantee the position accuracy and direction accuracy. The errors of the position at the impact point and the Euler angles rotated along the *x*, *y*, *z* axes are shown in Table 5.

**TABLE 4:** Joint angles according to the algorithm.

N°	$\theta_{1}(\text{°})$	$\theta_{2}(\text{°})$	$\theta_{3}(\text{°})$	$\theta_{4}(\text{°})$	$\theta_{5}(\text{°})$	$\theta_{6}(\text{°})$
1	−177.936,340	169.779,771	−58.752,296	91.309,992	125.711,066	32.945,428
2	27.198,690	−43.645,232	−88.014167	35.338,117	−56.159,879	−43.633,188
3	40.364,361	136.560,021	−124.683,465	154.842,073	16.853,445	84.766,356
4	26.260,466	175.113,664	−123.948,475	−95.208,397	107.305,427	−71.495,679

**TABLE 5:** Position and directional angle errors of the end-effector.

N°	Position Error of the End-Effector			Euler Angles Error of the End-Effector		
	x0(m)E	y0(m)E	x0(m)E	α0(rad)E	β0(rad)E	η0(rad)E
1	0	0	0	−9.98 × 10^–10^	4.07 × 10^–9^	−1.22 × 10^–9^
2	0	0	0	−1.57 × 10^–10^	1.28 × 10^–9^	−1.16 × 10^–10^
3	0	0	0	6.55 × 10^–10^	4.10 × 10^–10^	1.15 × 10^–9^
4	0	0	0	−2.83 × 10^–9^	−2.27 × 10^–9^	6.65 × 10^–11^

**FIGURE 5:**
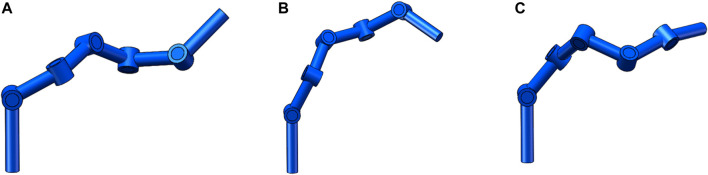
Schematic diagram of different joint angles of the robotic arm end-effector in the same state.

Tables 4 and 5 are the results. In Table 4, $\theta_{1}∼\theta_{6}$ indicates the joint angle 1∼joint angles 6 that satisfy the condition when the end-effector reaches the impact point after the robot arm moves. In Table 5, XE0,YE0,ZE0 indicates the new position of the robot arm end-effector after moving from the initial position. αE0,βE0,ηE0 indicates the Euler angles of rotation along the *x*, *y*, *z* axes after the robot arm end-effector moves from the initial position, respectively. The four sets of joint angles in Table 4 correspond to the positions and directions of the four sets of impact points in Table 3. Since there are often multiple sets of joint angles satisfying the conditions when the state of the end-effector of the robotic arm is certain, as shown in (a), (b), and (c) in Figure 5 (where the states of the end-effector of the robotic arm in (a), (b), and (c) are the same). Therefore, among the multiple sets of joint angles solved, a set of joint angles satisfying the range of joint angle values was selected as the final solution. By observing the position error of the robotic arm end-effector and the Euler angular error along the *x*,*y*,*z* axis rotation in the four sets of data in Table 5, we can find that the position error is always kept as 0, and the Euler angular error along the *x*,*y*,*z* axis rotation is between 10–11 and 10–9. Based on the error accuracy, we can initially judge that the algorithm can guarantee the position accuracy and orientation accuracy of the solution.

**FIGURE 6:**
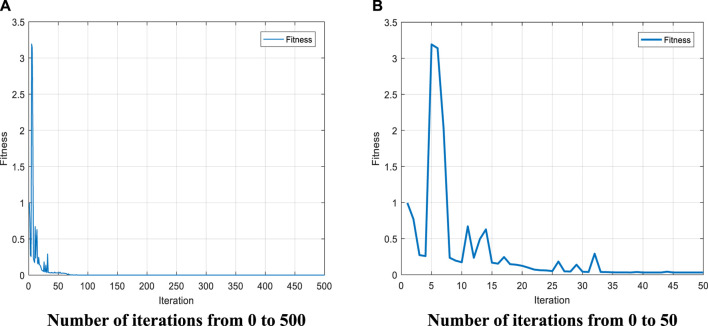
Plot of the variation of the fitness function with the number of iterations.

To evaluate the algorithm more comprehensively, images of the fitness function values and the number of iterations for the first set of joint angles were selected and are shown in Figures 6A,B, respectively. Figure 6A represents the fitness function values of the first set of joint angles with the number of iterations from 0 to 500. from Figure 6A, it can be seen that the fitness function reaches convergence when the number of iterations reaches about 100. Figure 6B represents the variation of the fitness function values for the first set of joint angles with the number of iterations from 0 to 50. It can be seen from Figure 6B that the fitness function value fluctuates up and down when the number of iterations is small, indicating that the algorithm has a strong search capability, while the fitness function value gradually converges while fluctuating, indicating that the fitness function value gradually approaches the global optimal solution. The combination of Figure 6A and Figure 6B shows that the algorithm has a certain search ability while converging.

### 6.2 Results Obtained for PUMA560 Robotic Arm

In order to better highlight the advantages of the present algorithm in terms of fast convergence and short time consumption while maintaining accuracy, the second experiment is conducted with the PUMA560 robotic arm as the research object and the paper (Deng and Xie, 2021; Yu et al., 2019) as the reference, by setting the same initial conditions for comparison experiments. The DH parameters of the PUMA560 robotic arm refer to the paper (Deng and Xie, 2021; Tian et al., 2020), and the specific parameter values are shown in Table 6. Experiment 2 was the same as experiment 1, and the initial position of the robotic arm end-effector as well as the Euler angles of rotation along the *x*, *y*, *z* axes were set, as shown in Table 6. The actual impact points of four robotic arm end-effectors were also set, and the positions of the end-effectors at this point as well as the Euler angles of rotation along the *x*, *y*, *z* axes were given, as shown in Table 6. The algorithms compared in Experiment 2 contain the algorithm proposed in this paper, the PSO algorithm and the QPSO algorithm. The relevant parameter settings of the different algorithms are shown in Table 6.

**TABLE 6:** Initial condition setting of PUMA560 robot arm.

Initial Conditions		Joint i	$\alpha_{\mathrm{i-1}}(\text{°})$	$a_{\mathrm{i-1}}\mathrm{(m)}$	$d_{i}\mathrm{(m)}$	$\theta_{i}(\text{°})$	Range of $\theta_{i}(\text{°})$
DH parameter		1	0	0	0	$\theta_{1}\left({}^{\circ}\right)$	−160–160
		2	−pi/2	0	0.1491	$\theta_{2}\left({}^{\circ}\right)$	−110–110
		3	0	0.4318	0	$\theta_{3}\left({}^{\circ}\right)$	−135–135
		4	−pi/2	0.0203	0.4331	$\theta_{4}\left({}^{\circ}\right)$	−266–266
		5	pi/2	0	0	$\theta_{5}\left({}^{\circ}\right)$	−100–100
		6	-pi/2	0	0	$\theta_{6}\left({}^{\circ}\right)$	−266–266
Initial position and Euler angles		**Initial position of the end-effector**			**Initial Euler angles of the end-effector**		
		${\text{x}}_{\text{0}}\left(m\right)$	${\text{y}}_{\text{0}}\left(m\right)$	${\text{z}}_{\text{0}}\left(m\right)$	$\alpha_{\text{0}}\left(rad\right)$	$\beta_{\text{0}}\left(rad\right)$	$\eta_{\text{0}}\left(rad\right)$
		0.452	0.149	−0.433	0	0	-0.0175
Given the position and the Euler angle	**N°**	**Position of end impact point**			**Euler angles of end impact point**		
		x0(m)E	y0(m)E	x0(m)E	y0(m)E	x0(m)E	y0(m)E
	1	0.1,527,529	0.3,001,222	0.2,861,892	−2.706,829	0.7,287,091	0.6,550,095
	2	0.04,577,256	0.2,597,535	0.1,975,715	−2.895,429	0.4,328,479	1.501,387
	3	−0.08,576,319	0.31616	0.154,809	−2.797,244	0.1,117,431	2.215,782
	4	-0.1,647,316	0.4,121,032	0.2,377,258	−2.513,781	−0.06,674,747	2.950,499
Algorithm parameter	**Parameters**		**This paper**		**PSO**	**APSO**	**QPSO**
	$c_{1}$		1.4		1.4	1.2	-
	$c_{2}$		1.4		1.4	1.2	-
	$\omega_{\max}$		0.9		0.9	0.9	-
	$\omega_{\min}$		0.7		-	0.7	-
	$\beta_{0}$ and $\beta_{1}$		-		-	-	0.5 and 1

In Table 6, ${\text{x}}_{\text{0}},{\text{y}}_{\text{0}},{\text{z}}_{0}$ denotes the initial position of the robotic arm end-effector, $\alpha_{0},\beta_{0},\eta_{0}$ denotes the initial Euler angles of the robotic arm end-effector rotated along the x, y, and *z* axes, respectively. XE0,YE0,ZE0 denotes the new position of the robotic arm end-effector after moving from the initial position, and αE0,βE0,ηE0 denotes the Euler angles of the robotic arm end-effector rotating along the *x*, *y*, *z* axes after moving from the initial position, respectively. Table 7 show the six joint angles corresponding to the impact point obtained by the algorithm proposed in this paper, the PSO algorithm and the QPSO algorithm, respectively. The errors of different algorithms regarding the position and Euler angles of rotation along the *x*, *y*, *z* axes of the impact point are shown in Table 8. Considering that the differences in the initial conditions of different algorithms affect the fairness of the results, the number of iterations is set to 500, and the number of particle swarms is 150.

**TABLE 7:** The six joint angles corresponding to the impact points obtained by different algorithms.

Algorithm	N°	$\theta_{1}(\text{°})$	$\theta_{2}(\text{°})$	$\theta_{3}(\text{°})$	$\theta_{4}(\text{°})$	$\theta_{5}(\text{°})$	$\theta_{6}(\text{°})$
This paper	1	139.147,212	−78.742,965	2.298,743	223.577,471	−47.319,473	−89.781,627
	2	−153.871,884	−72.479,104	44.314,641	−102.235,380	−13.594,398	229.782,832
	3	−133.020650	−37.399,877	59.160,356	176.205,721	42.374,612	−74.592,745
	4	−64.557,618	−59.510,032	75.432,529	−148.731,249	49.034576	−81.522,956
PSO	1	139.145,321	−78.729,959	2.302,142	−136.418,272	−47.307,830	−89.791,711
	2	−153.873,831	−72.478,187	44.301,555	−102.268,096	−13.599,806	229.818,471
	3	−133.017595	-37.394,194	59.156,632	−183.792,886	42.377,145	−74.591,502
	4	−73.090507	−69.438,932	78.661,376	27.906,941	−44.210,988	91.724,240
QPSO	1	9.607,712	−20.610,209	69.598,100	−235.293,713	12.944,303	212.995,170
	2	−118.0051	−40.9856	49.9457	−2.6864	−39.7447	−204.2855
	3	113.9081	73.6642	34.8149	167.6001	90.6805	161.9327
	4	124.0405	45.9792	77.2999	146.3037	98.7763	134.9092

**TABLE 8:** The errors in position and Euler angles of impact points by different algorithms.

Algorithm	N°	Position Error of the End-Effector			Euler Angles Error of the End-Effector		
		x0(m)E	y0(m)E	z0(m)E	α0(rad)E	β0(rad)E	η0(rad)E
This paper	1	0	0	0	−1.70 × 10^–9^	−1.76 × 10^–9^	2.03 × 10^–9^
	2	0	0	0	2.07 × 10^–9^	−6.62 × 10^–9^	5.09 × 10^–11^
	3	0	0	0	2.19 × 10^–10^	1.29 × 10^–8^	4.57 × 10^–10^
	4	0	0	0	−2.35 × 10^–9^	8.56 × 10^–9^	1.58 × 10^–9^
PSO	1	−3.4 × 10^–4^	2.83 × 10^–5^	−1.46 × 10^–4^	2.24 × 10^–5^	−1.01 × 10^–4^	−1.90 × 10^–4^
	2	−7.40 × 10^–4^	−1.64 × 10^–5^	1.53 × 10^–4^	−2.49 × 10^–5^	2.89 × 10^–4^	7.41 × 10^–6^
	3	−5.78 × 10^–5^	−2.05 × 10^–5^	−2.01 × 10^–4^	−4.03 × 10^–6^	1.45 × 10^–5^	4.50 × 10^–6^
	4	0.065	-0.019	0.097	−8.09 × 10^–4^	0.013	−0.0019
QPSO	1	4.66	−1.00	−4.02	−1.00	−1.00	−1.02
	2	17.72	−1.02	−5.41	−1.00	−0.99	−1.02
	3	−11.46	−0.93	−6.45	−1.00	−1.24	−1.00
	4	−6.80	−0.82	−4.35	−1.00	2.13	−0.98

**FIGURE 7:**
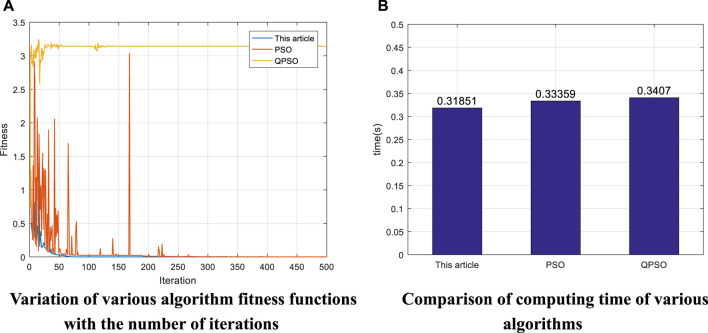
Comparison of the results of various algorithms for the puma560 robot arm.

Tables 7 and 8 represent the results. In Table 7, $\theta_{1}∼\theta_{6}$ indicates the joint angle 1∼joint angle 6 that satisfy the condition when the end-effector reaches the impact point after the robot arm moves. In Table 8, XE0,YE0,ZE0 indicates the new position of the robot arm end-effector after moving from the initial position. αE0,βE0,ηE0 indicates the Euler angles of rotation along the *x*, *y*, *z* axes after the robot arm end-effector moves from the initial position, respectively. By comparing the error in Table 8, it can be found that the position error of the algorithm proposed in this paper is 0, and the orientation error is between 5.09 × 10^–11^ and 1.29 × 10^–8^. The position error of the conventional PSO algorithm is between -0.019 and 0.097, and the orientation error is between -0.019 and 0.013. The position error of the QPSO algorithm is between -11.46 and 17.72, and the orientation error is between -1.24 and 2.13. The comparison shows that the proposed algorithm can guarantee higher position and orientation accuracy compared with the traditional PSO algorithm and QPSO algorithm. The joint angles obtained in Table 7 are all within the range of values, so it can be determined that there is a set of joint angles that allow the end-effector to reach the impact point, i.e., the error comparison of each algorithm is meaningful. To further illustrate the advantages of the algorithms in terms of convergence and operation speed, the first set of data in Table 6 was selected in Experiment 2 to compare the variation of the fitness function with the number of iterations and the operation time in each algorithm, and the results are shown in Figures 7A,B, respectively.

Figure 7A summarizes the variation of the fitness function with the number of iterations in various algorithms. It can be seen that the algorithm proposed in this paper converges steadily when the number of iterations reaches about 50. In contrast, the PSO algorithm fluctuates more and does not converge significantly, and the algorithm fluctuates more when it is close to convergence and the number of iterations reaches about 175, indicating that the algorithm is prone to fall into the local optimum and thus misses the optimal solution, resulting in poor solution accuracy. Compared with the first two algorithms, the QPSO algorithm is unable to maintain convergence. Through comparison, it can be found that the algorithm proposed in this paper converges faster and at the same time ensures stable convergence. Figure 7B shows the time required for various algorithms to run the first set of data in Table 8. The histogram shows that the algorithm proposed in this paper has the shortest operation time of 0.31851s, while the PSO algorithm and QPSO algorithm have an operation time of 0.33359 and 0.3407s, respectively. By comparing several factors such as end-effector position accuracy, direction accuracy, convergence of the algorithm and operation time, the algorithm proposed in this paper has a good performance.

### 6.3 Results Obtained for the Seven-Degree-of-Freedom Robotic Arm

In order to reflect the wide applicability of the present algorithm, the third experiment is conducted with a seven-degree-of-freedom robotic arm as the object of study, and the paper (Dereli S, Koker R 2019) is used as a reference for comparison experiments by setting the same initial conditions. Where the DH parameters of the seven-degree-of-freedom robotic arm are set as shown in the paper (Dereli and Koker, 2020; Li et al., 2019a; Li et al., 2019b; Li et al., 2019c). The specific DH parameter table is shown in Table 9. Experiment 3 set up a set of initial positions of the robotic arm end-effectors as well as the Euler angles of rotation along the *x*, *y*, *z* axes, as shown in Table 9. The actual impact point of a set of robotic arm end-effectors was also set, and the position of the end-effectors at this point as well as the Euler angles of rotation along the *x*, *y*, *z* axes were given, as shown in Table 9. The algorithms compared in Experiment 3 contain the algorithm proposed in this paper, the PSO algorithm, and the QPSO algorithm.

**TABLE 9:** Initial condition setting of Seven degrees of freedom robot arm.

Initial Conditions	Joint i	$\alpha_{\mathrm{i-1}}(\text{°})$	$a_{\mathrm{i-1}}\mathrm{(m)}$	$d_{i}\mathrm{(m)}$	$\theta_{i}(\text{°})$	Range of $\theta_{i}(\text{°})$
DH parameter	1	−pi/2	0	0.5	$\theta_{1}\left({}^{\circ}\right)$	−180–180
	2	pi/2	0.2	0	$\theta_{2}\left({}^{\circ}\right)$	-90–30
	3	−pi/2	0.25	0	$\theta_{3}\left({}^{\circ}\right)$	−90–120
	4	pi/2	0.3	0	$\theta_{4}\left({}^{\circ}\right)$	−90–90
	5	−pi/2	0.2	0	$\theta_{5}\left({}^{\circ}\right)$	−90–90
	6	0	0.2	0	$\theta_{6}\left({}^{\circ}\right)$	−90–90
	7	0	0.1	0.05	$\theta_{7}\left({}^{\circ}\right)$	−30–90
Initial position and Euler angles	**Initial position of the end-effector**			**Initial Euler angles of the end-effector**		
	${\text{x}}_{\text{0}}\left(m\right)$	${\text{y}}_{\text{0}}\left(m\right)$	${\text{z}}_{\text{0}}\left(m\right)$	$\alpha_{\text{0}}\left(rad\right)$	β0(rad)E	$\eta_{\text{0}}\left(rad\right)$
	1.250	0.050	0.500	0	0.0175	0
Given the position and the Euler angle	**Position of end impact point**			**Euler angles of end impact point**		
	x0(m)E	y0(m)E	z0(m)E	α0(rad)E	β0(rad)E	η0(rad)E
	−0.5839	−0.7154	−0.5027	0.5977	0.7025	−1.69

In Table 9, ${\text{x}}_{\text{0}},{\text{y}}_{\text{0}},{\text{z}}_{0}$ denotes the initial position of the robotic arm end-effector, $\alpha_{0},\beta_{0},\eta_{0}$ denotes the initial Euler angles of the robotic arm end-effector rotated along the x, y, and *z* axes, respectively. XE0,YE0,ZE0 denotes the new position of the robotic arm end-effector after moving from the initial position, and αE0,βE0,ηE0 denotes the Euler angles of the robotic arm end-effector rotating along the *x*, *y*, *z* axes after moving from the initial position, respectively. The six joint angles corresponding to the impact points obtained by different algorithms are shown in Table 10. The errors of different algorithms regarding the position and Euler angles of rotation along the *x*, *y*, *z* axes of the impact points are shown in Table 11. Considering that the differences in the initial conditions of different algorithms affect the fairness of the results, the number of iterations is set to 500, and the number of particle swarms is 150.

**TABLE 10:** Joint angles according to different algorithms.

Algorithm	$\theta_{1}\left({}^{\circ}\right)$	$\theta_{2}\left({}^{\circ}\right)$	$\theta_{3}\left({}^{\circ}\right)$	$\theta_{4}\left({}^{\circ}\right)$	$\theta_{5}\left({}^{\circ}\right)$	$\theta_{6}\left({}^{\circ}\right)$	$\theta_{7}\left({}^{\circ}\right)$
This article	41.2034	5.3333	48.9559	49.5050	48.3672	43.5106	42.0882
PSO	64.2282	4.7456	23.8832	48.4828	51.2608	62.2661	23.7729
QPSO	69.7373	2.6296	32.7903	57.5889	42.0689	15.3571	63.6031

**TABLE 11:** Position and orientation errors according to different algorithms.

Algorithm	Position Error of End-Effector			Euler Error of the End-Effector		
	x0(m)E	y0(m)E	z0(m)E	α0(rad)E	β0(rad)E	η0(rad)E
This article	0	0	0	0	0	0
PSO	7.53 × 10^–4^	0.0010	−0.0025	−0.0010	5.69 × 10^–4^	−2.96 × 10^–4^
QPSO	−1.77	−2.42	−1.42	2.78	−0.58	−1.62

**FIGURE 8:**
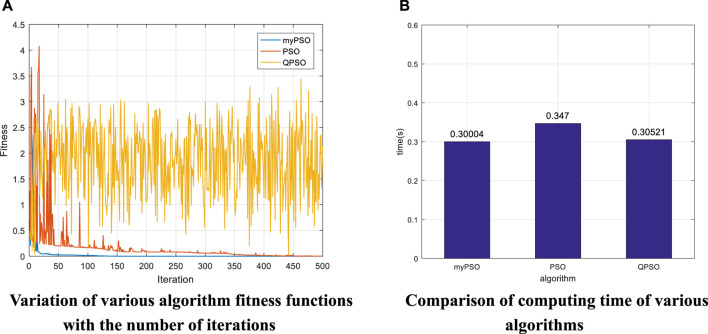
Comparison of the results of various algorithms for seven-degree-of-freedom robotic arms.

Tables 10 and 11 represent the results. In Table 10, $\theta_{1}∼\theta_{7}$ indicates the joint angle 1∼joint angles 7 that satisfy the condition when the end-effector reaches the impact point after the robot arm moves. XE0,YE0,ZE0 indicates the new position of the robot arm end-effector after moving from the initial position. αE0,βE0,ηE0 indicates the Euler angles of rotation along the *x*, *y*, *z* axes after the robot arm end-effector moves from the initial position, respectively. Through the error in Table 11, it can be found that the position error of the algorithm proposed in this paper is 0 and the orientation error is 0. The position error of the conventional PSO algorithm is between -0.0025 and 0.0010, and the orientation error is between -0.0010 and 5.69 × 10^–4^. The position error of the QPSO algorithm is between -2.42 and -1.42, and the orientation error is between -1.62 and 2.78. The comparison shows that when the object is a seven-degree-of-freedom robot arm, the proposed algorithm can still guarantee higher position and orientation accuracy compared with the traditional PSO algorithm and QPSO algorithm. The joint angles obtained in Table 10 are all within the range of values, so it can be determined that the joint angles enable the end-effector to reach the impact point, i.e., the comparison of the errors of each algorithm is meaningful. To further illustrate the advantages of the algorithms in terms of convergence and operation speed, the data in Table 9 were selected for Experiment 3 to compare the variation of the fitness function with the number of iterations and the operation time in each algorithm, and the results are shown in Figures 8A,B, respectively.

Figure 8A summarizes the variation of the fitness function with the number of iterations in various algorithms. It can be seen that the algorithm proposed in this paper converges faster than the other two algorithms, converging at less than 100 iterations. The fact that the value of the fitness function does not fluctuate after convergence indicates that the convergence is more stable. In contrast, the PSO algorithm converges slowly, reaching about 350 iterations before convergence, and there are still small fluctuations when convergence is near. Compared with the first two algorithms, the QPSO algorithm is unable to maintain convergence. The comparison shows that the proposed algorithm can still maintain fast convergence and stable convergence compared to the other two algorithms when the object of study is a seven-degree-of-freedom robot arm. Figure 8B shows the running time of various algorithms. The histogram shows that the algorithm proposed in this paper has the shortest operation time of 0.30004s, while the operation time of PSO and QPSO algorithms are 0.347 and 0.30521s respectively. By replacing the experimental object and comparing several factors such as end-effector position accuracy, direction accuracy, convergence of the algorithm and operation time, the algorithm proposed in this paper can still maintain good performance, which can show that the algorithm proposed in this paper has great potential in solving the inverse kinematics problem of multi-degree-of-freedom robotic arm.

## 6.4 Discussions

Robot inverse kinematics solutions are of great importance because they are the basis for the subsequent study of robot path planning and control. The existing robot inverse kinematics solutions, such as analytical and geometric methods, can be applied to a limited number of scenarios, and they are mainly used for robotic arms that satisfy the “Pieper” criterion and have analytical solutions. The intelligent algorithm represented by particle swarm algorithm is becoming a more promising and exploitable means of solving robot inverse kinematics with high accuracy, short time and wide range of application. In this paper, the traditional particle swarm algorithm is used as the basis, and its inertia weights as well as the initial population are mainly optimized. Based on the characteristics that large inertia weights are suitable for global search and small weights are suitable for local search, an adaptive weighting strategy is proposed in this paper. Adjusting the inertia weights according to the change of the fitness function can effectively reduce the probability of the function value falling into the local optimum. According to Figures 7A, 8A, it can be seen that when the fitness function is close to convergence, the traditional PSO algorithm still has a large undulation phenomenon, i.e., the situation that the value of the fitness function falls into a local minimum, while by introducing the adaptive weight strategy, the undulation phenomenon at convergence is effectively reduced, as shown in the blue curve of Figure 7A. In this paper, we propose a condition setting based on the limit joints. Firstly, the maximum and minimum values of each joint angle are determined according to the range of joint angles, then the position factor k is introduced (through several experiments, k = 0.5 is finally taken), and finally the velocity factor is determined by the position factor k as well as the maximum joint angle. Due to the introduction of the position coefficient k in the velocity factor, a reasonable reference standard is provided for the initialization of position and velocity. At the same time, the speed of the algorithm is improved by introducing the condition setting of the limit joints before the iteration instead of continuously performing the boundary detection during the iteration. Finally, an exponential product form modeling method (POE) based on spinor theory is chosen. Compared with the traditional DH modeling method, the spinor approach describes the motion of a rigid body as a whole and avoids the singularities that arise when described by a local coordinate system. The above three experiments confirm the advantages of the algorithm proposed in this paper in terms of solution accuracy, operation speed, convergence of the algorithm and applicability. Experiment 1 takes a general six-degree-of-freedom industrial robotic arm as the research object, and sets up four sets of robotic arm end-effector impact point positions and Euler angles of rotation along the *x*, *y*, *z* axes to verify the algorithm. Table 5 shows that the final end-effector position error solved by the algorithm is 0 and the orientation error is 10^–11^–10^–9^, which can initially show that the algorithm can guarantee the position accuracy and orientation accuracy of the end-effector. Meanwhile, the variation of the fitness function with the number of iterations in Figures 6A,B of Experiment 1 can preliminarily show that the algorithm has convergence. Experiment 2 takes the PUMA560 robotic arm as the research object and compares the algorithm proposed in this paper with a variety of algorithms by replacing the data taking the same verification method as Experiment 1. The error in Table 8 show that the position error of the proposed algorithm is 0 and the maximum orientation error is 1.29 × 10^–8^, while the lowest position error of other algorithms is -1.64 × 10^–5^ and the lowest orientation error is 4.5 × 10^–6^. The data in Figure 7B show that the running time of the proposed algorithm is 0.31851s, while the shortest running time of other algorithms is 0.33359s. The curve in Figure 7A shows that the proposed algorithm converges faster and more stably than the other algorithms. Through various comparisons, it can be found that the proposed algorithm has higher accuracy of position and direction solving, faster operation speed, and faster and more stable convergence than other algorithms. Experiment 3 takes a seven-degree-of-freedom manipulator as the research object and sets up one group of robot arm end-effector impact point positions as well as Euler angles of rotation along the *x*, *y*, *z* axes to verify the algorithm. The error in Table 11 shows that the position error of the proposed algorithm is 0 and the orientation error is 0, while the lowest position error of the other algorithms is 7.53 × 10^–4^ and the lowest orientation error is -2.69 × 10^–4^. The data in Figure 8B shows that the running time of the proposed algorithm is 0.30004s, while the shortest running time of the other algorithms is 0.30521s. The curve in Figure 8A shows that the proposed algorithm converges faster and more stably than the other algorithms. Through various comparisons, it can be found that the proposed algorithm can maintain its own advantages for different research objects and has wide applicability. This paper also summarizes the improved PSO algorithm applied to the inverse kinematics solution of robotic arm, and compares different algorithms in terms of both position error and solution time, as shown in Table 12.

**TABLE12:** Comparison with some studies in the literature.

Research	Robotic Arm	Technique Used	Comparative Technique	
(Mustafa A and Kerim C 2016)	4DOF	QPSO	GA	
		6.51e-06	3.96e-04	Position Error(m)
		1.96	17.53	Solution Time(s)
Liu et al. (2021a)	6DOF	PLPSO	DE	
		2.5918e−15	1.0798e−07	Position Error(m)
		4.4982	8.3929	Solution Time(s)
Dereli and Koker. (2018)	7DOF	Random IW-PSO	Global-Local Best IW-PSO	
		6.20e−03	3.64e−03	Position Error(m)
		1.6	1.2	Solution Time (s)
(Serkan D, Rasit K, et al., 2019)	7DOF	QPSO	PSO	
		2.775e−17	6.719e−03	Position Error(m)
		0.2319	0.4498	Solution Time(s)

## 7 Conclusion

In this paper, the algorithm is verified and compared in terms of solution accuracy, operation time and convergence through three experiments. Experiment 1 takes a general six-degree-of-freedom industrial robotic arm as the research object, and sets up four groups of robotic arm end-effector impact point positions as well as postures. By bringing the relevant parameters of the robotic arm, the range of joint angle values, the initial postures and positions of the robotic arm end-effectors into the algorithm, the joint angles that meet the conditions are obtained, and finally the actual robotic arm end-effector positions and postures are obtained. The convergence of the algorithm is also initially illustrated by the variation of a selected set of fitness functions with the number of iterations. The final position error brought into the algorithm is 0, and the orientation error interval is 10^–9^∼10^–11^, which preliminarily illustrates that the algorithm can guarantee a certain accuracy of position and orientation solution. Experiment 2 compares the algorithm proposed in this paper with the traditional particle swarm algorithm (PSO) and quantum particle swarm algorithm (QPSO) in terms of solution accuracy, operation time and convergence, using the PUMA560 robotic arm as the research object. In which the experimental approach is consistent with Experiment 1, the position error of the algorithm proposed in this paper is 0, the maximum direction error is 1.29 × 10^–8,^ and the operation time of a set of data is 0.31851s. The minimum position error of the other two algorithms is -1.64 × 10^–5^, the minimum direction error is -4.03 × 10^–6^, and the operation time of a set of data is 0.33359s. At the same time, by comparing the changes of the fitness function with the number of iterations in a set of data, it can be found that the algorithm proposed in this paper converges faster and more stably than other algorithms. Finally, through various comparisons, it can be found that the algorithm proposed in this paper can guarantee high accuracy of position and direction solving, faster solving speed, and more stable and faster convergence. Experiment 3 takes a seven-degree-of-freedom robotic arm as the object of study, sets up a group of robotic arm end-effector impact point positions and postures and repeats the operation steps of experiment 2, in which the position error of the proposed algorithm is 0, the orientation error is 0, and the operation time is 0.30004s. The minimum position error of the other two algorithms is 7.53 × 10^–4^, the minimum orientation error is −2.96 × 10^–4^, and the minimum operation time is 0.30521s. At the same time, by comparing the changes of the fitness function with the number of iterations, we can find that the algorithm proposed in this paper still maintains the advantages of stable and fast convergence. By replacing different experimental objects and comparing with various algorithms, it can be found that the algorithm proposed in this paper can maintain its advantages in solution accuracy, operation time and convergence while having strong applicability, which indicates that the algorithm has greater potential in solving the inverse kinematics of multi-degree-of-freedom robotic arm. In the future, we will start from improving the stability of the particle swarm algorithm, and strive to find the ideal solution with less number of trials. In addition, the study of robot arm motion control will also be carried out in the follow-up work.

## Data Availability

The original contributions presented in the study are included in the article/Supplementary Material, further inquiries can be directed to the corresponding authors.
